# How sequencing technology shapes our understanding of river water microbiomes and resistomes: a comparative study

**DOI:** 10.1128/aem.01723-25

**Published:** 2025-09-19

**Authors:** Jialin Hu, J. Chris Blazier, Anna Gitter, Lucas F. Gregory, Terry J. Gentry

**Affiliations:** 1 Department of Soil and Crop Sciences, Texas A&M University 199056, College Station, Texas, USA; 2 Texas A&M Institute for Genome Sciences and Society, Texas A&M University 14736, College Station, Texas, USA; 3 School of Public Health, University of Texas Health Science Center at Houston 53622, El Paso, Texas, USA; 4 Texas Water Resources Institute, Texas A&M AgriLife Research, College Station, Texas, USA; University of Delaware, Lewes, Delaware, USA

**Keywords:** aquatic microbiome, antibiotic resistance, virulence factors, metagenomics, Nanopore

## Abstract

River ecosystems are vital for supporting biodiversity and supplying freshwater, but are increasingly impacted by microbial pollution, including the spread of antibiotic resistance genes (ARGs), which poses growing public health concerns. While high-throughput sequencing technologies have advanced our ability to study microbial communities and resistomes, their varying capabilities and biases require comparative analysis. In this study, we compared three sequencing approaches—Illumina 16S rRNA amplicon, Illumina shotgun metagenomics, and Oxford Nanopore-based long-read metagenomics—to profile microbial communities, ARGs, and virulence factors (VFs) in 48 river water samples. All methods identified Proteobacteria and Actinobacteria as dominant phyla, but substantial differences emerged at finer taxonomic levels. Long-read metagenomics and 16S data showed greater consistency at the genus level, while Illumina metagenomics differed, detecting more potential pathogens and fewer native freshwater taxa. For ARG and VF profiling, unassembled Illumina data yielded higher diversity and abundance, but assembled Illumina data showed comparable results to long-read metagenomics data in terms of dominant genes and host associations. Although Illumina provides high sensitivity, the use of short reads and associated assembly limitations can compromise functional accuracy. In contrast, long-read metagenomics facilitates gene-level resolution and direct host linkage, providing a more comprehensive understanding of environmental microbiomes. Our findings highlight the strengths and limitations of each method and support Oxford Nanopore technology (ONT)-based long-read metagenomic sequencing as a cost-effective and informative tool for high-resolution taxonomic and functional analysis of complex environmental samples.

**IMPORTANCE:**

Accurate characterization of microbial communities and their functional traits, such as antibiotic resistance, is essential for evaluating water quality and associated public health risks. However, the selection of sequencing methods can substantially influence the detection and interpretation of microbial community composition and functional potential in environmental samples. By directly comparing amplicon, short-read metagenomic, and long-read metagenomic sequencing across 48 freshwater samples collected across different sites and time points, this study builds upon earlier work that typically focused on only two methods or less complex communities. It provides a comparative evaluation of three widely used sequencing approaches, demonstrating how methodological differences affect the resolution and reliability of taxonomic and functional profiling in complex environmental microbiomes. By highlighting the strengths and limitations of each platform, these findings enhance our understanding of how sequencing strategy shapes environmental microbiome analyses and contributes to evidence-based method selection in environmental microbiology and antimicrobial resistance monitoring.

## INTRODUCTION

River ecosystems play a vital role in the global water cycle and biodiversity conservation, offering unique habitats for diverse animals, plants, and microorganisms. They are critical freshwater sources, supporting ecological functions and meeting human demands for clean water and irrigation (1, 2). However, microbial pollution and the growing prevalence of the resistome (i.e., collections of antibiotic resistance genes [ARGs]) in freshwater ecosystems have generated significant concern due to their potential risks for public health (3, 4). While antibiotic resistance occurs naturally, increasing anthropogenic activities, such as urbanization, agriculture, and wastewater discharge, have accelerated the spread of antibiotic-resistant bacteria, ARGs, and even antibiotic-resistant pathogenic bacteria in aquatic environments. This has intensified global concerns about waterborne diseases and associated health risks posed by the increasing antimicrobial resistance. Notably, at least 1.7 billion people globally rely on drinking water sources polluted by fecal microbes (World Health Organization, 2023), while waterborne pathogens are responsible for an estimated 7.15 million illnesses and at least $3 billion in healthcare costs annually in the United States (5). Furthermore, bacterial antibiotic resistance directly caused nearly 1.27 million deaths globally in 2019 (6). These issues are exacerbated by agricultural runoff, urbanization, wastewater discharge, and leaching from nearby farms, which introduce a variety of pollutants into freshwater ecosystems (4). Therefore, detecting, understanding, and mitigating these risks are critical for protecting public health and ensuring the sustainability of freshwater resources.

High-throughput sequencing technologies have enabled comprehensive profiling of microbial communities across diverse environments (7). Amplicon sequencing (e.g., 16S rRNA, 18S rRNA, and ITS) remains a widely used approach for examining microbial diversity and structure in various environments (7–10) and even as an advanced microbial source tracking technology (11, 12), but it has limited species-level resolution and lacks functional insight (13, 14). In contrast, Illumina-based short-read metagenomic sequencing enables direct whole-genome profiling of microbial communities and has been increasingly applied to functional characterization, including detection of ARGs and potential pathogens (15–20). However, it can still face challenges in taxonomic resolution. For example, conserved marker genes, such as 16S rRNA, may fail to assemble, and genome reconstruction can be uneven, as some taxa may be underrepresented or fragmented due to low abundance or genomic complexity. Taxonomic classifiers like Kraken2 rely on k-mer matches; however, Illumina short reads may not contain enough unique k-mers to confidently assign taxonomy at the species or strain level, especially for closely related organisms. Furthermore, its reliance on short reads limits the ability to directly link functional genes to their microbial hosts and complicates complete genome assembly in complex environmental samples (21). In recent years, long-read metagenomic sequencing using Oxford Nanopore technology (ONT) has gained significant attention for its capacity to generate long reads, with average lengths ranging from several kilobases to over 100 kilobases (22), which enhances the precision and efficiency of directly linking functional genes to their microbial hosts (23). However, its lower accuracy (~95%) compared to the Illumina platform (~99.9%) has greatly limited its widespread application (24). With advancements in the ONT sequencing platform and development of improved base-calling algorithms, its accuracy has significantly improved (25). To date, the application of long-read metagenomics for detecting ARGs and potential pathogens in freshwater ecosystems remains underutilized. Furthermore, although some studies have compared long- and short-read metagenomic sequencing technologies, most focused on only two approaches and were limited to individual microbial genomes (26, 27), viral genomes sequencing (28, 29), or taxonomic resolution in relatively low-complexity microbiomes, such as mock communities, clinical samples, and other host-associated environments (30–32). Additionally, some comparisons have been limited to 16S rRNA gene sequencing data rather than true metagenomic analyses (24, 33). Direct comparisons involving all three approaches (amplicon, short-read metagenomics, and long-read metagenomics) for taxonomic analysis are rare. Even fewer studies have assessed their relative performance in functional profiling, particularly in environmental samples with more complex microbial communities and substantial temporal and spatial variability, such as river water microbiomes. While high-throughput sequencing technologies have revolutionized microbiome research, they differ in resolution, biases, and suitability depending on the research objective. This highlights the importance of selecting the most appropriate method for each study.

Therefore, this study aimed to compare how three widely used high-throughput sequencing methods—16S rRNA gene amplicon sequencing (hereafter amplicon sequencing), Illumina shotgun metagenomic sequencing (hereafter short-read metagenomics), and ONT long-read metagenomic sequencing (hereafter long-read metagenomics)—differ in their ability to characterize river water microbiomes and resistomes, including microbial community composition, ARGs, and virulence factors (VFs). We applied these methods to 48 river water samples collected across 12 time points from four monitoring sites along the Lavaca River in coastal Texas. In addition, we used ONT long reads and assembled Illumina contigs to directly associate ARGs and VFs with their potential microbial hosts, enabling deeper insights into gene-host relationships beyond abundance profiles. The Lavaca River watershed is a predominantly rural area characterized by dispersed agricultural activities, including rangeland, pasture, and row crops. By critically comparing these sequencing approaches, our study reveals their respective strengths and limitations in resolving microbial and resistome dynamics in freshwater ecosystems, providing valuable insights to inform methodological choices in environmental microbiology studies and antimicrobial resistance monitoring.

## MATERIALS AND METHODS

### Sample collection and DNA extraction

**Fig 1:**
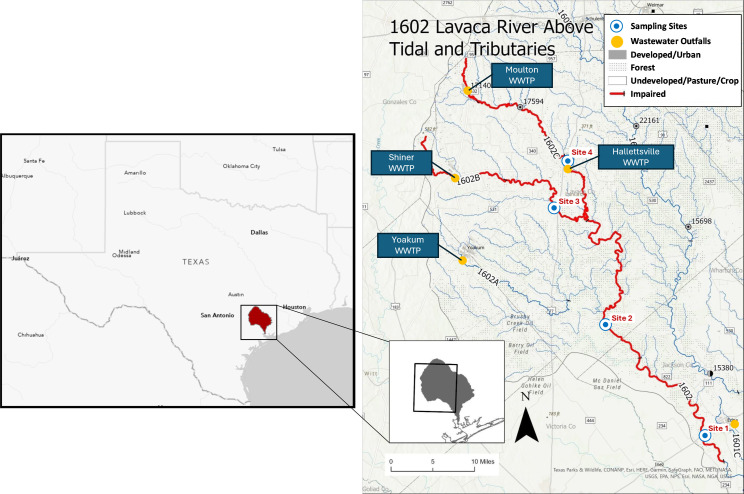
Overview of the location of sampling sites. Figure adapted from *Lavaca Basin 2021 Water Quality Update* and *Lavaca Basin 2024 Watershed Summary*, with permission from Lavaca-Navidad River Authority (LNRA).

Sampling was conducted at four monitoring sites in the Lavaca River watershed, Texas, at 12 time points between February 2023 and March 2024, yielding a total of 48 samples (Table S1). Sites 1 (28°57'36.9"N, 96°41'11"W) and 2 (29°9'24"N, 96°52'30"W) were situated along the midstream section of the Lavaca River, while Site 3 (29°21'39.24"N, 96°58'27.48"W) was located on the tributary Rocky Creek, downstream of a wastewater treatment plant near the city of Shiner. Site 4 (29°26'35"N, 96°56'39"W) was positioned upstream on the Lavaca River, close to the city of Hallettsville (Fig. 1). River water samples were collected following the Texas Commission on Environmental Quality Surface Water Quality Monitoring Procedures (RG-415, Chapter 5). For sites where water depth exceeded 0.5 m, samples were collected from the centroid of flow at approximately 0.3 m below the surface. For shallower sites (≤0.5 m depth), samples were collected at approximately one-third of the total water depth from the surface. Bottles were submerged carefully to minimize surface contamination and turbulence. Water samples were collected in 1 L sterilized plastic bottles. Samples were transported to the laboratory in an ice-filled cooler on the day of collection. A 100 mL volume of each water sample was filtered through Supor 0.2 µm pore-size membrane filters (47 mm diameter, Pall Corporation, MI, USA). The filters were placed in 47 mm petri dishes and stored at −80°C until DNA extraction.

DNA extraction for ONT-based long-read metagenomic sequencing was performed in our lab using the ZymoBIOMICS DNA Miniprep Kit (Zymo Research, CA, USA), following the manufacturer’s instructions. Prior to extraction, the filter was cut into small pieces to enhance extraction efficiency. Extracted DNA was submitted to the Texas A&M Institute for Genome Sciences and Society and stored at −20°C until library preparation for sequencing. For Illumina-based amplicon and short-read metagenomic sequencing, the water filters were submitted to Zymo Research, where DNA extraction was performed using the same kit to ensure consistency across sequencing platforms. To evaluate potential contamination during library preparation and sequencing, both positive and negative controls obtained from Zymo Research were included in the Illumina sequencing run. A blank extraction sample was used as the negative control, while the ZymoBIOMICS Microbial Community Standard served as the positive control. These controls were processed and sequenced alongside the study samples. The taxa detected in the positive control closely matched its expected theoretical composition, and no taxa were detected in the negative control, demonstrating that no external contamination occurred during sample processing or sequencing (Fig. S1).

### Amplicon, short-read, and long-read metagenomic sequencing

The 16S rRNA gene amplicon sequencing was performed by ZymoBIOMICS Targeted Sequencing Service. DNA library preparation utilized the Quick-16S NGS Library Prep Kit (Zymo Research, CA, USA). The V3–V4 region (~466 bp) of the 16S rRNA gene was amplified using primers 341F (a mixture of 5′-CCTACGGGDGGCWGCAG-3′ and 5′-CCTAYGGGGYGCWGCAG-3′, 17 bp) and 806R (5′-GACTACNVGGGTMTCTAATCC-3′, 24 bp). The final library was sequenced on an Illumina Nextseq platform with a P1 reagent kit (600 cycles), incorporating a 30% PhiX spike-in.

ONT-based long-read metagenomic sequencing was performed by the Texas A&M Institute for Genome Sciences and Society. DNA libraries were performed using the Rapid PCR Barcoding Kit (SQK-RPB004, Oxford Nanopore Technologies, Oxford Science Park, United Kingdom) with 5 ng DNA input, following the manufacturer’s protocol. The sequencing was carried out on a PromethION flow cell. Illumina short-read metagenomic sequencing was conducted by Zymo Research Shotgun Metagenomic Sequencing Service. DNA libraries were prepared using the Illumina DNA Library Prep Kit (Illumina, San Diego, CA) with up to 500 ng DNA input following the manufacturer’s protocol using unique dual-index 10 bp barcodes with Nextera adapters (Illumina, San Diego, CA). All libraries were pooled in equal concentrations. The final pool was quantified using qPCR and TapeStation (Agilent Technologies, Santa Clara, CA), and sequenced on an Illumina NovaSeq platform (150 bp × 2).

### Bioinformatics

The raw amplicon sequencing data targeting the V3–V4 region of the 16S rRNA gene were processed using QIIME2 (v 2023.2). Amplicon sequence variants were inferred using the DADA2 plugin (qiime dada2 denoise-paired). Primer sequences were removed by trimming 17 bases from the 5′ end of the forward reads and 21 bases from the reverse reads. Reads were truncated at 280 bp (forward) and 260 bp (reverse) based on quality profiles to remove low-quality tails. Chimeric sequences were identified and removed using the “consensus” method. Singletons were removed before taxonomic analysis. The taxonomy classification was performed using a Naive Bayes classifier trained on the Greengenes database (v13.8) targeting the V3–V4 region, with reference sequences limited to 300–600 bp in length. Classification was carried out using the classify-sklearn function in QIIME2 with a confidence threshold of 0.7, which is the default value recommended for balanced taxonomic resolution.

The ONT long-read metagenomic sequencing yielded an average of 9.5 million raw reads per sample, ranging from 6.4 to 15.8 million reads before trimming (Table S2). The adapter trimming of raw reads was performed using Porechop (v0.2.4). The -discard_middle setting was used to eliminate reads with internal adapters, which have a chance of being chimeric and thus causing cross-contamination among samples multiplexed on the same flow cell. An average of 9.4 million clean reads per sample (range: 6.3 to 15.7 million reads) with an average read length of 3,139 bp was obtained (Table S2). Taxonomic classification was conducted on clean reads using Kraken2 (v2.0.9) (34). To minimize the impact of extremely low-abundant and potentially unreliable taxa, species-level taxa with a clade-level read count ≤1 (i.e., singleton species) in the Kraken2 report files were excluded from downstream analyses. ARGs and VFs were identified using ABRicate (v1.0.0), a genomic screening tool that scans contigs against reference databases of resistance and virulence genes. Analyses were conducted using the Comprehensive Antibiotic Resistance Database (CARD) and Virulence Factor Database (VFDB), applying thresholds of ≥80% coverage and identity.

The Illumina short-read metagenomic sequencing produced an average of 173.5 million raw reads per sample, ranging from 119.1 million to 340.3 million reads (Table S2). The quality control of short-read metagenomic sequencing data was performed using FastQC (35), and trimming was carried out with Trimmomatic v0.39 (36), applying a sliding window of 4 bp with a quality cutoff of 15. Reads containing “N” bases or shorter than 100 bp were removed. Clean paired-end reads were merged into interleaved FASTA format using Seqtk (v1.3) (https://github.com/lh3/seqtk). After trimming, an average of 160.6 million clean reads per sample was obtained, ranging from 111.4 million to 318.0 million reads (Table S2). The samples S1-T9 and S2-T9, which had the highest number of clean reads, were selected to test the impact of duplication issues arising during library preparation and sequencing (optical duplication) on the accuracy of the revealed microbial community composition. Two reference-free deduplication methods, Clumpify and Dedupe, were applied to remove duplicates independently. Taxonomic analysis was conducted using Kraken2. To reduce the influence of extremely low-abundance or potentially unreliable taxa, singleton species were excluded from downstream analyses. ARGs and VFs were determined by aligning clean reads against CARD and VFDB databases using DIAMOND (v2.1.9) with an e-value cutoff of ≤10^−5^, identity ≥90%, and bit score ≥100. Illumina short reads were also assembled using MEGAHIT (v1.2.9) with a minimum contig length threshold of 500 bp. Across all 48 river water samples, assemblies yielded an average of 887,743 contigs per sample, with maximum contig lengths ranging from 41,273 to 1,111,326 bp. The total length of assembled contigs per sample ranged from 422.55 to 1423.45 million bp, representing an average of 1.80% (ranging from 0.95% to 2.99%) of the total clean reads. The assembly statistics are summarized in Table S3. Taxonomy classification of the assembled Illumina contigs was performed using Kraken2. Species identified from single assembled contigs were retained, as these long, quality-filtered contigs provide adequate sequence information for k-mer-based taxonomic assignment. ARGs and VFs were subsequently detected in the assembled contigs using ABRicate against CARD and VFDB with thresholds of ≥80% coverage and identity.

The abundances of ARGs and VFs detected in assembled contigs were normalized as contigs per million (CPM), i.e., the number of contigs annotated with a specific gene category divided by the total number of assembled contigs, multiplied by one million.

For long-read and unassembled short-read metagenomic data, ARGs and VFs abundances were normalized using the equation:


$$RPKBB=\;\frac{GeneNumReads}{\frac{GeneLength}{1000}\times \frac{TotalBasePairs}{1000000000}}$$

where RPKBB denotes reads per kilobase per billion base pairs; GeneNumReads is the number of reads mapped to the specific gene; GeneLength is the length of the specific gene in the database (bp); and TotalBasePairs represents the total number of base pairs of the sequences. RPKBB normalization accounts for gene length and sequencing depth, correcting the inherent bias that longer genes attract more reads and enabling accurate cross-sample comparisons of gene abundance.

To compare alpha diversity metrics (observed species, Pielou’s evenness, and Shannon index) and beta diversity (based on Bray-Curtis distance) across sequencing methods (amplicon, long-read metagenomics, and short-read metagenomics), diversity analyses were carried out at the species level in QIIME2 (v2023.2). To assess the sufficiency of sequencing depth for resistome analysis, rarefaction curves based on ARG counts were generated for the top four samples with the highest number of clean reads from both long-read and short-read metagenomic data sets.

### Statistical analysis

The Shapiro-Wilk test was conducted to assess the normality of the data. If data fit a normal distribution, one-way analysis of variance was used to evaluate whether group means were significantly different. If data did not meet normality, the Kruskal-Wallis test was used instead. Permutational multivariate analysis of variance (PERMANOVA) based on Bray-Curtis distance matrix was used to evaluate the dissimilarity of microbial communities across sites and time points. Principal coordinates analysis (PCoA) was conducted in R using the packages vegan (v 2.5-7) (37), phyloseq (v 1.38.0) (38), and ggplot2 (v 3.3.5) (39) based on Bray-Curtis distance matrix. The Mantel test was used to assess similarities among beta diversity distance metrics derived from amplicon, short-read, and long-read metagenomic sequencing data.

To link ARGs and VFs to their microbial hosts, taxonomic information obtained from Kraken2 was assigned to ONT-based long reads or assembled Illumina contigs that were identified by ABRicate as carrying ARGs or VFs. These direct gene-taxon associations were then visualized using network analysis in Gephi (v 1.0) with the Fruchterman-Reingold layout.

## RESULTS

### Taxonomic classification based on three sequencing platforms

For metagenomic sequencing, on average, approximately 68.0% of high-quality ONT long reads and 62.6% of Illumina short reads were classified into specific taxa (Table 1). Among the classified long reads, 92.5% were identified as bacteria, 6.9% as eukaryota, and 0.5% collectively as archaea and viruses. Similarly, for classified short reads, 93.4% were identified as bacteria, 6.5% as eukaryota, and 0.2% were accounted together by archaea and viruses (Table 1).

**TABLE 1:** Summary statistics of clean reads across 48 river water samples that could be classified into superkingdoms by Kraken2

	% of classified reads	% of superkingdom within classified reads			
		Bacteria	Eukaryota	Archaea	Viruses
Nanopore long-read metagenomic sequencing					
Min	54.7	72.8	2.6	0.1	0.1
Max	80.2	97.1	26.5	1.0	0.6
Mean	68.0	92.5	6.9	0.2	0.3
CV (%)	8.3	6.1	80.0	63.8	39.4
Illumina short-read metagenomic sequencing					
Min	50.9	88.0	3.9	0.1	0.0
Max	77.5	96.1	11.7	0.3	0.1
Mean	62.6	93.4	6.5	0.2	0.0
CV (%)	8.76	1.5	1.4	0	49.5

Taxonomic classification at the phylum and genus levels was compared across the data obtained from amplicon, long-read, and short-read metagenomic sequencing based on the 48 river water samples. Amplicon data identified the highest number of taxa at higher taxonomic levels, detecting 59 phyla and 1,504 genera, compared to long-read metagenomic (36 phyla, 1,223 genera) and short-read metagenomic data (24 phyla, 719 genera). However, at the species level, long-read metagenomics detected the most species (3,377), followed by short-read metagenomics (2,173) and amplicon data (1,561).

**Fig 2:**
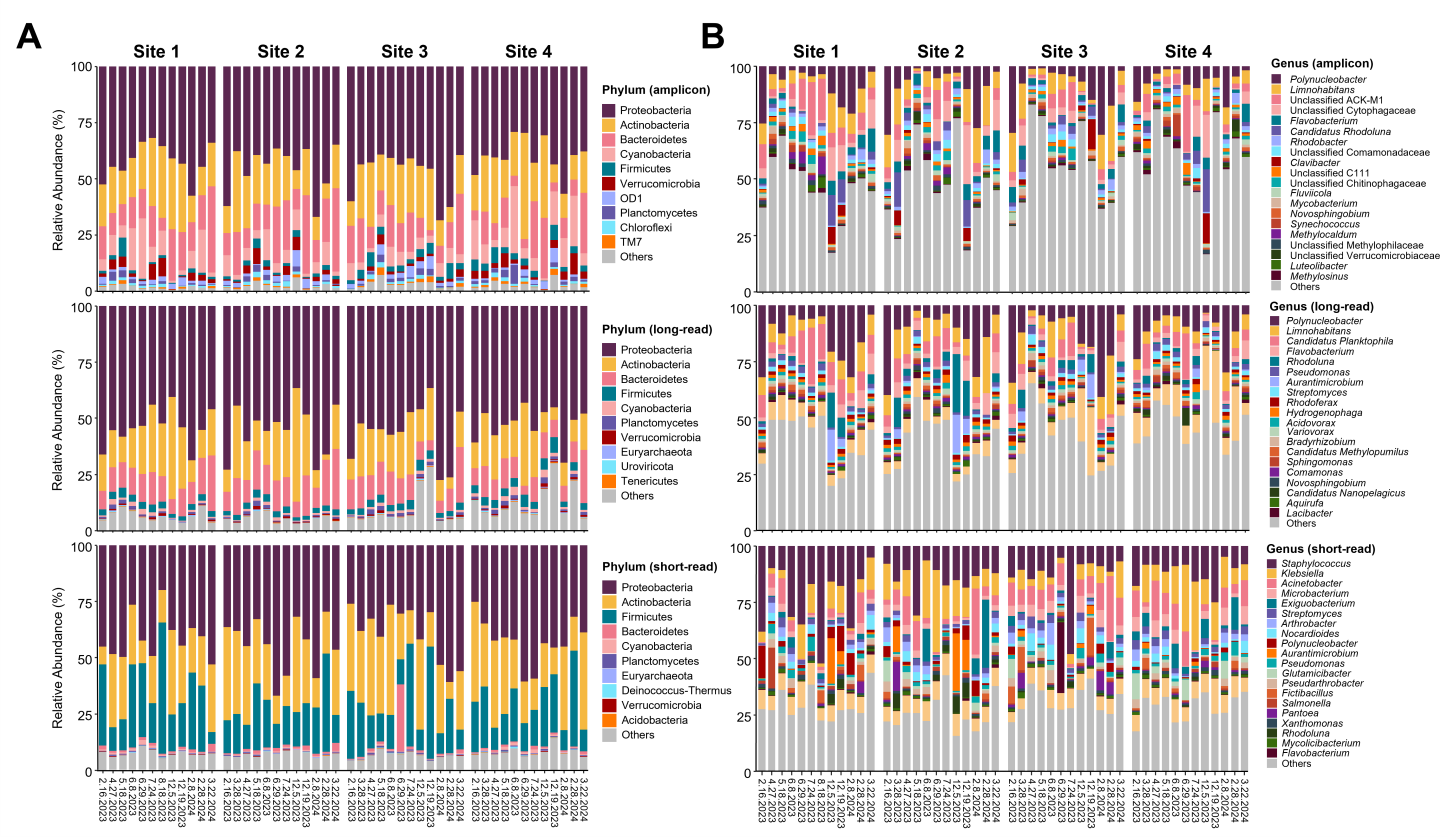
Microbial community composition of Lavaca River water samples at phylum (**A**) and genus (**B**) levels based on 16S rRNA amplicon sequencing, Oxford Nanopore long-read metagenomic sequencing, and Illumina short-read metagenomic sequencing.

Proteobacteria and Actinobacteria were the predominant phyla in the river water microbial communities across all sequencing methods. Proteobacteria comprised approximately 43.8%, 54.4%, and 41.6% of the community based on amplicon, long-read metagenomic, and short-read metagenomic data, respectively, while Actinobacteria accounted for 20.3%, 18.5%, and 26.6%, respectively. The third most abundant phylum differed between methods, with Bacteroidetes identified in amplicon data (15%) and long-read metagenomics (12.4%), while Firmicutes were identified in short-read metagenomics (21.6%) (Fig. 2A). At the genus level, the top five most abundant bacterial genera identified by amplicon data and long-read metagenomics included *Polynucleobacter*, *Limnohabitans*, *Candidatus Planktophila*, an unidentified genus of *Cytophagaceae*, *Flavobacterium*, and *Rhodoluna*. In contrast, the genera identified by short-read metagenomics differed substantially, with *Staphylococcus*, *Klebsiella*, *Acinetobacter*, *Microbacterium*, and *Exiguobacterium* being the top five abundant bacterial genera. *Polynucleobacter*, the most abundant genus based on amplicon data and long-read metagenomics, only ranked ninth in short-read metagenomics (Fig. 2B). Additionally, for the two selected short-read metagenomic data sets used to assess the potential impact of duplication issues, Dedupe calculated duplication rates of approximately 19%, while Clumpify reported rates of about 13% for all duplicates and 7% for optical duplicates. Despite the occurrence of duplicate reads, the microbial community composition at both the phylum and genus levels exhibited similar trends across non-deduplicated, optical duplicate-removed, and all duplicate-removed data (Fig. S2).

### Alpha and beta diversity of microbial communities

The alpha diversity indices (observed species, Pielou’s evenness, and Shannon index) obtained from amplicon and long-read metagenomic data varied significantly with sampling time (*P* < 0.05) but not with sampling sites. In contrast, Pielou’s evenness derived from short-read metagenomic data was significantly influenced by both sampling time and sites (*P* < 0.05) (Table S4). Notably, all three sequencing methods revealed a similar temporal trend, with higher alpha diversity values in spring and lower in summer and winter across all sampling sites (Fig. S3). Furthermore, long-read metagenomics detected significantly higher alpha diversity metrics compared to short-read metagenomics (*P* < 0.001), and amplicon data exhibited the lowest species richness (Fig. S3).

**Fig 3:**
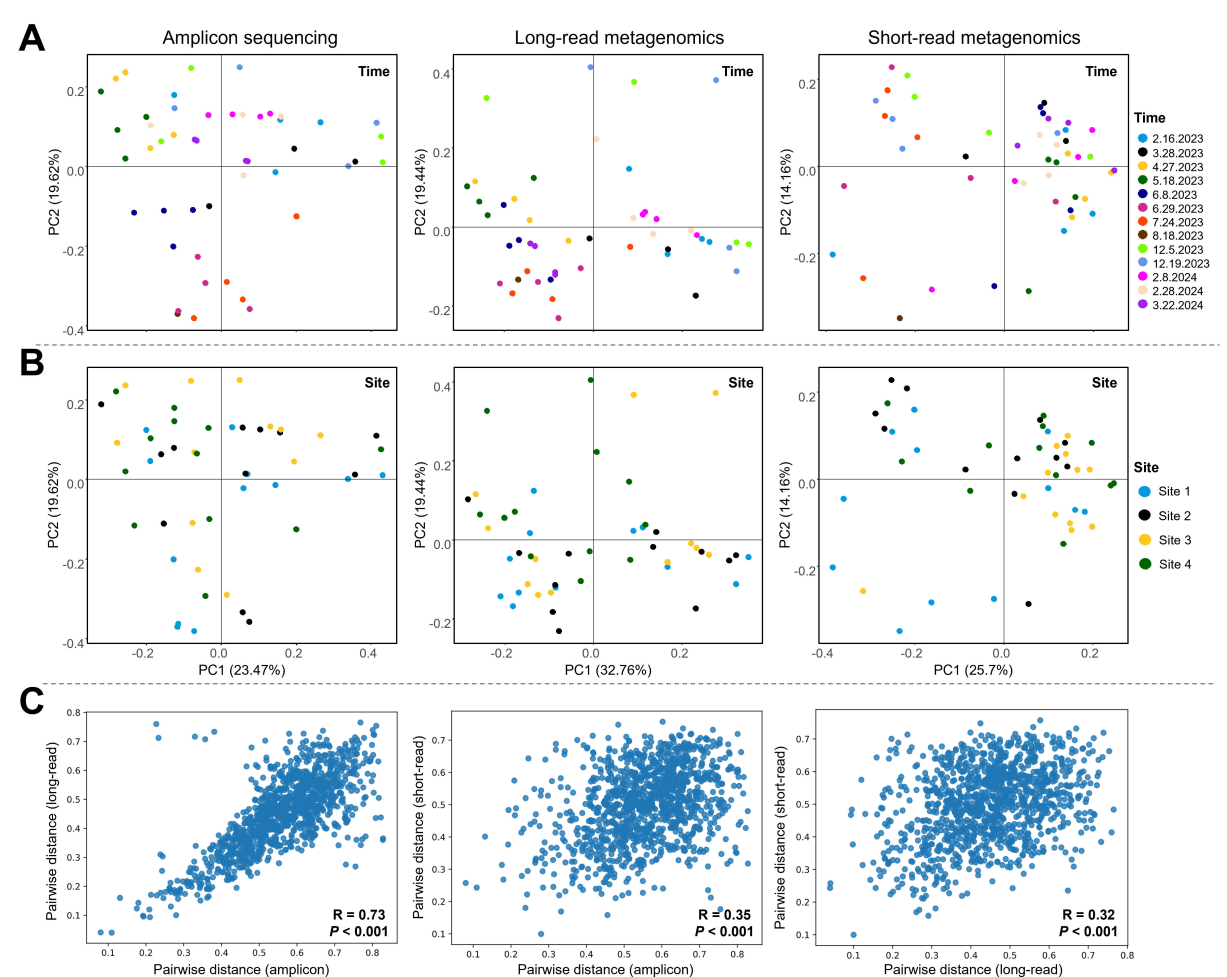
PCoA based on the Bray-Curtis dissimilarity index at the species level, illustrating variations in river water microbial community structures across (**A**) sampling times and (**B**) sampling sites. (**C**) Mantel test results showing the correlation/similarity among microbial community structures identified by 16S rRNA amplicon sequencing, Oxford Nanopore long-read metagenomic sequencing, and Illumina short-read metagenomic sequencing.

PCoA based on Bray-Curtis distances revealed significant variations in microbial community composition across sampling times, independent of the sequencing method used (PERMANOVA, *P* < 0.001) (Fig. 3A). Microbial communities in river water showed no significant differences among sampling sites when analyzed using amplicon or long-read metagenomic data. However, short-read metagenomics revealed significant variations in microbial communities across the sampling sites (*P* = 0.008), with Site 1 differing significantly from Site 3 (*P* = 0.017) and Site 4 (*P* = 0.022), and Site 2 being different from Site 3 (*P* = 0.026) (Fig. 3B).

The Mantel test revealed a strong and statistically significant positive correlation between the beta diversity distance matrices of amplicon sequencing data and long-read metagenomic data (R = 0.73, *P* < 0.001), while showing weaker correlations with the short-read metagenomic data (R = 0.35 for 16S; R = 0.32 for Nanopore) (Fig. 3C). This test confirmed the higher similarity in the composition and temporal dynamics of river water microbial communities between amplicon and long-read metagenomic data, as well as their divergence from short-read metagenomics.

### Profiling of ARGs and VFs

**Fig 4:**
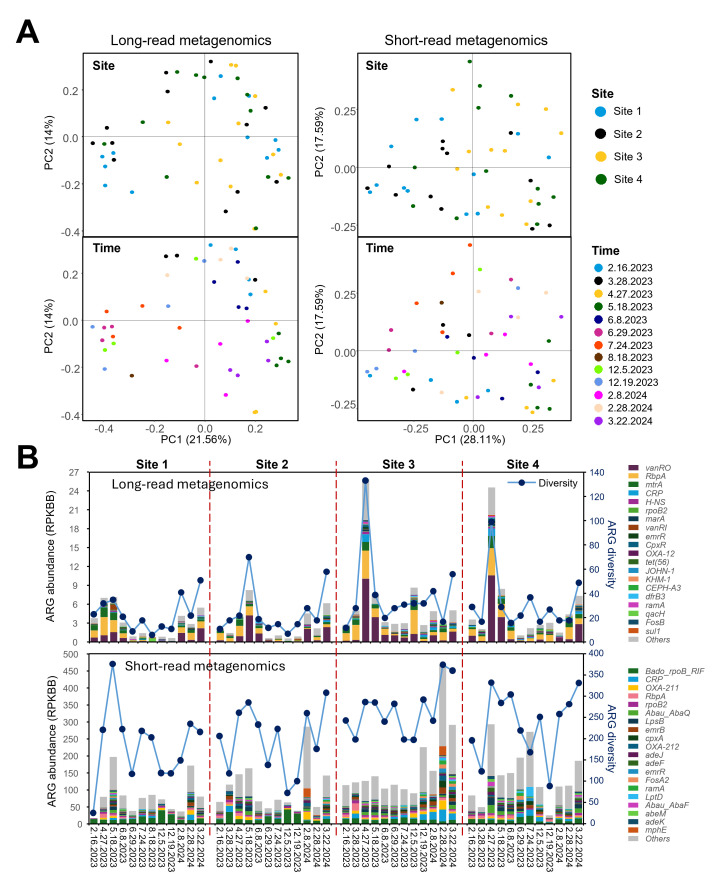
(**A**) Distribution patterns of ARGs in river water samples across different sampling times and locations, as determined by Nanopore long-read and Illumina short-read metagenomic sequencing. (**B**) Comparison of the normalized abundance and diversity of ARGs in river water samples detected using Nanopore long-read and Illumina short-read metagenomic sequencing.

The detection of ARGs and VFs was conducted by aligning clean reads from long-read and short-read metagenomics against the CARD and VFDB databases, respectively. Temporal variation (i.e., sampling time) significantly influenced ARG profiles regardless of sequencing method (PERMANOVA, *P* < 0.001 for both), while spatial differences (i.e., sampling site) had no significant effect. This was further supported by PCoA, which showed that samples clustered primarily by time rather than by site (Fig. 4A). The abundance of ARGs showed distinct trends between sequencing methods. Long-read metagenomic data showed no significant variation across the four sampling sites but significant differences across sampling times (Kruskal-Wallis test, *P* < 0.01), with highest abundance in spring and lowest in summer (Fig. 4B). Conversely, short-read metagenomic data showed significant spatial differences (*P* < 0.05) but no temporal variation, with the highest ARG abundance at Site 3 (Rocky Creek, a tributary) and the lowest at Sites 1 and 2 (midstream in the Lavaca River) (Fig. 4B).

A notable difference in ARG diversity was observed between the sequencing methods: a total of 371 ARGs resistant to 20 different antibiotics were identified from long-read metagenomics, whereas 986 ARGs resistant to 23 antibiotics were detected from short-read metagenomics, with 280 ARGs overlapping between these two methods and five overlapping within the top 20 most abundant ARGs. While the ARG-based rarefaction curves for both long-read and short-read metagenomic data sets remain unsaturated, the short-read data sets are nearing saturation more closely than the long-read data sets (Fig. S4). The diversity of ARGs was affected by both spatial (*P* < 0.05) and temporal factors (*P* < 0.01) regardless of sequencing method, with higher diversity observed at Site 3 and in spring (March, April, and May) while lower at Site 1 and in early winter (December) (Fig. 4B). It is worth noting that a strong positive correlation was detected between diversity and abundance of ARGs for both long-read (R = 0.824, *P* < 0.001) and short-read data sets (R = 0.792, *P* < 0.001). ARGs were categorized by antibiotic resistance classes, which also varied by method. For long-read metagenomic data, the top resistance classes were glycopeptide, rifamycin, and multidrug, whereas for short-read data, the top classes were multidrug, beta-lactam, and peptide (Fig. S5). The most abundant ARGs detected in long-read metagenomics were *vanRO*, *rbpA*, and *mtrA*, conferring resistance to glycopeptides, rifamycin, and multidrug antibiotics, respectively. In contrast, the most abundant ARGs identified through short-read metagenomics were *Bado_rpoB_RIF*, *CRP*, and *OXA-211*, associated with rifampicin, multidrug, and beta-lactam resistance, respectively (Fig. 4B).

**Fig 5:**
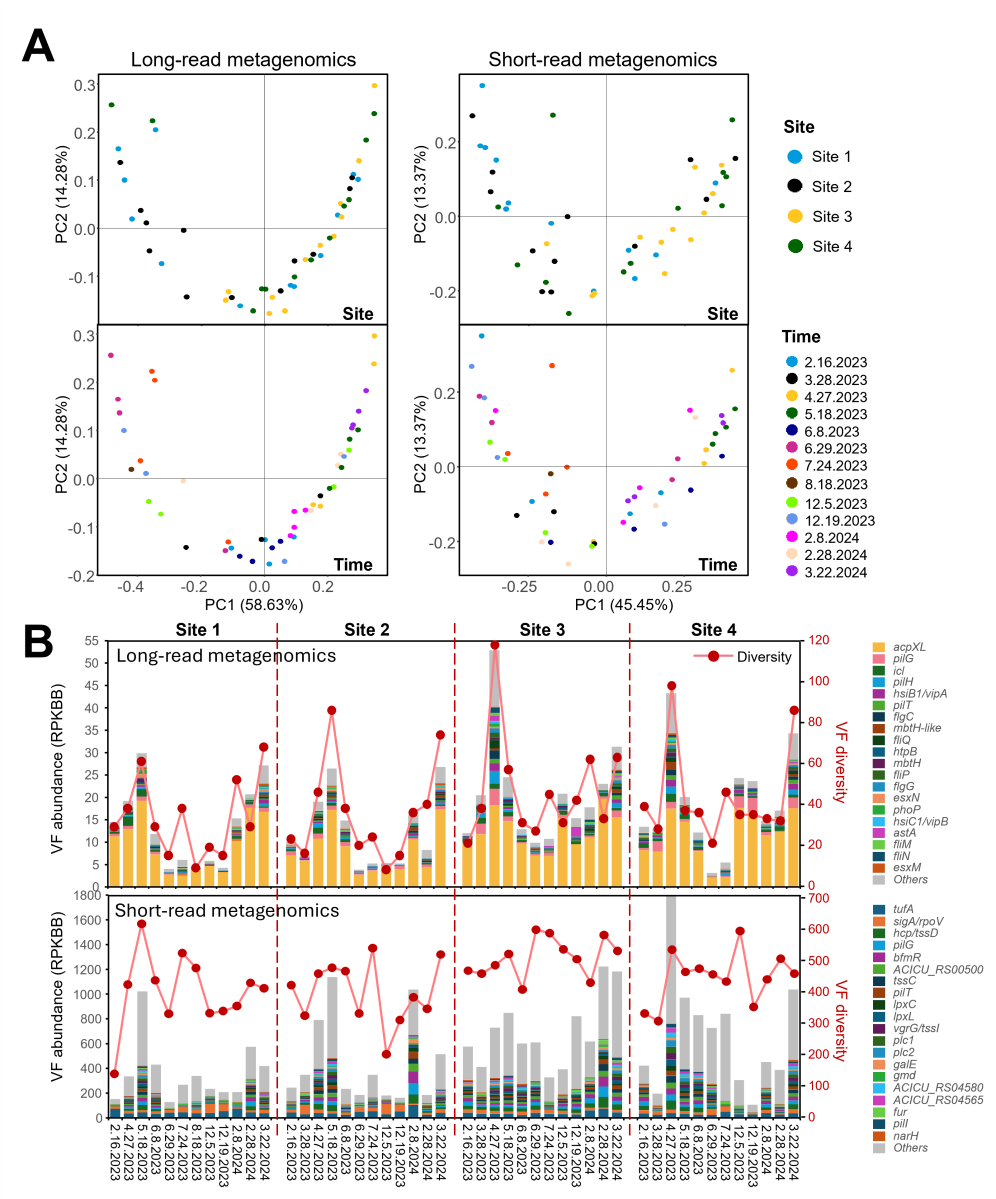
(**A**) Distribution patterns of VFs in river water samples across different sampling times and locations, as determined by Nanopore long-read and Illumina short-read metagenomic sequencing. (**B**) Comparison of the normalized abundance and diversity of VFs in river water samples detected using Nanopore long-read and Illumina short-read metagenomic sequencing.

Similarly, the profiles of VFs varied significantly with sampling time, independent of sequencing method (*P* < 0.001). Spatial variation affected VF profiles detected by short-read metagenomics (*P* < 0.05) but not by long-read data. Specifically, short-read data revealed significant differences in the profiles of VFs among sampling sites, with Site 1 differing significantly from Site 3 (*P* = 0.003) and Site 4 (*P* = 0.047) and Site 2 being different from Site 3 (*P* = 0.016), which was visualized by PCoA (Fig. 5A). Regarding VF abundance, long-read data indicated no significant spatial differences but significant temporal variation (*P* < 0.001), with the highest values in spring and the lowest in summer (Fig. 5B). Short-read data, however, showed significant spatial differences (*P* < 0.05) but no temporal variation, with the highest VF abundance observed at Site 3 and the lowest at Site 1 (Fig. 5B).

Difference in VF diversity detected by long-read and short-read metagenomics was also evident: long-read data identified 267 VFs, whereas short-read data detected 1,313 VFs, with 213 VFs shared between the two methods and only two shared with the top 20 abundant VFs. The diversity of VFs based on short-read data was influenced solely by sampling site (*P* < 0.05), with Site 3 exhibiting higher diversity compared to other sites. In contrast, VF diversity based on long-read data was significantly affected by sampling time (*P* < 0.001), with greater diversity observed during the spring months (March, April, and May) (Fig. 4B). Furthermore, significant positive correlation was detected between diversity and abundance of VFs for both long-read (R = 0.76, *P* < 0.001) and short-read data (R = 0.564, *P* < 0.001). The most abundant VFs detected differed by sequencing method. Long-read metagenomics predominantly identified *acpXL*, *pilG*, *icl*, and *pilH*, while short-read metagenomics highlighted *tufA*, *sigA*/*rpoV*, *hcp*/*tssD*, and *pilG* as the most abundant VFs (Fig. 5B).

Out of approximately 42.61 million contigs assembled from Illumina short-read metagenomic data, 2,860 were annotated as carrying ARGs and 2,432 as carrying VFs. Among them, 253 ARGs were identified, of which 220 were also detected in the unassembled Illumina short-read data and 169 were shared with long-read data (Fig. S6A). Similarly, 184 VFs were detected in the assembled contigs, with 160 overlapping with unassembled short-read data and 134 shared with long-read data (Fig. S6B).

**Fig 6:**
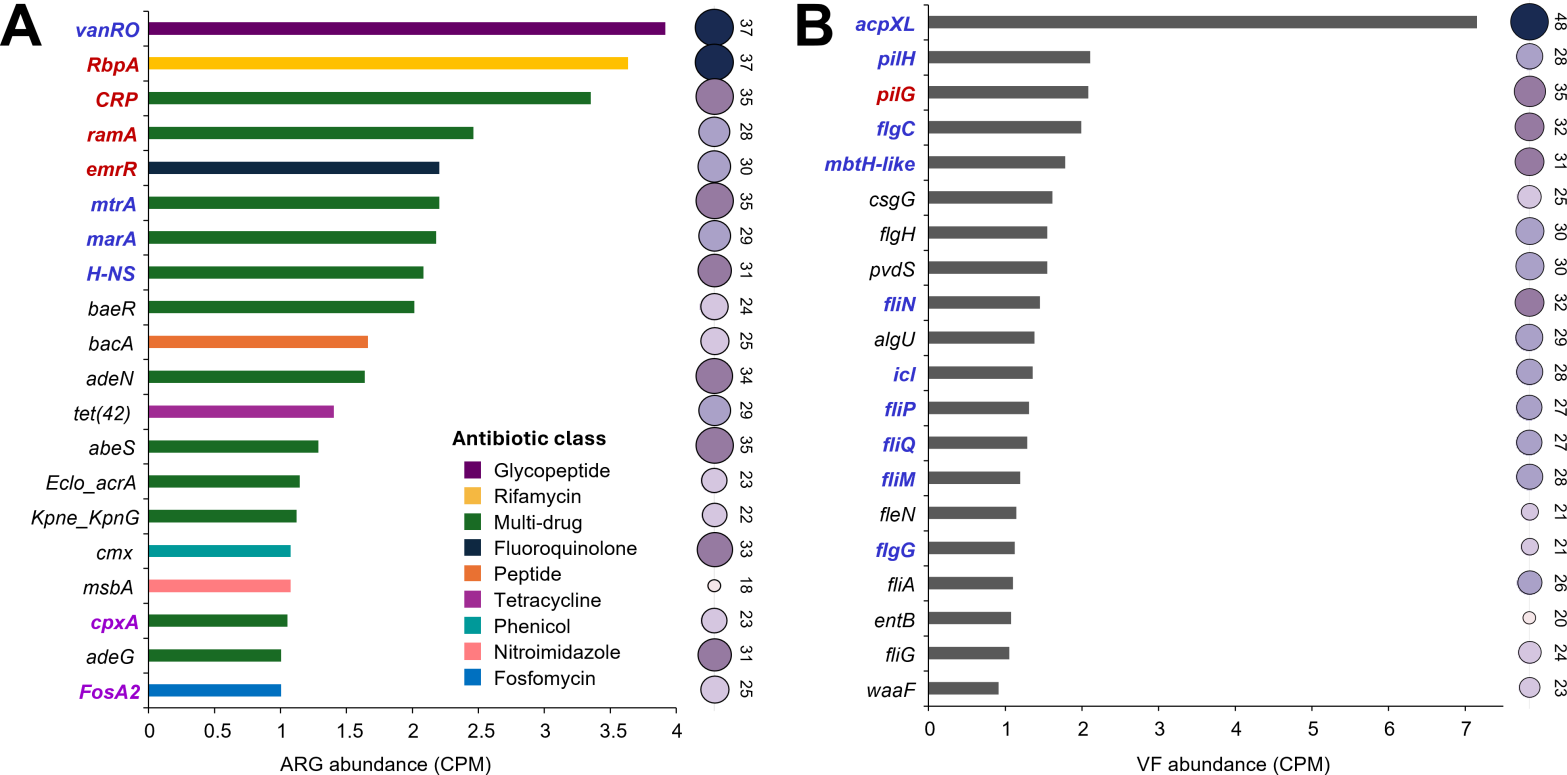
The top 20 most abundant (**A**) ARGs and (**B**) VFs detected from assembled Illumina contigs. Bar plots represent normalized abundance (CPM). The numbers next to the bubbles indicate the number of samples (out of 48) in which each gene was detected, and the size and color of bubbles were used for visualization. Genes labeled in blue, purple, and red indicate they were also detected among the top 20 most abundant in Nanopore long-read data, unassembled Illumina short-read data, and both data sets, respectively.

Among the top 20 most abundant ARGs detected in the assembled Illumina contigs, the majority were classified as multidrug resistance genes, and 16 of the 20 were present in at least half of the samples (≥24 out of 48) (Fig. 6A). Comparison with the top 20 abundant ARGs detected in long-read metagenomic data showed an overlap of eight ARGs, including seven that were also ranked within the top 10 in both data sets (*vanRO*, *RbpA*, *CRP*, *emrR*, *mtrA*, *marA*, and *H-NS*). Notably, *vanRO* and *RbpA* were the two most abundant ARGs identified across both data sets. In contrast, comparison with unassembled Illumina short-read data revealed only six overlapping ARGs within the top 20, with just two shared among the top 10 (Fig. 4B; Fig. 6A).

A similar trend was observed for VFs. Among the top 20 most abundant VFs detected from the assembled Illumina contigs, most were found in at least half of the samples, with *acpXL* and *pilH* being the most abundant (Fig. 6B). When compared to the ONT-based long-read metagenomic data, 11 of the top 20 VFs overlapped, including five that were also shared within the top 10 in both data sets (*acpXL*, *pilH*, *pilG*, *flgC*, and *mbtH*). In contrast, only one VF from the top 20 in the contigs overlapped with those from the unassembled Illumina reads (Fig. 5B; Fig. 6B). Together, these findings showed greater consistency between the assembled Illumina results and ONT long-read metagenomic data in identifying abundant ARGs and VFs, compared to unassembled Illumina reads.

### Linking ARGs and VFs to microbial taxa

**Fig 7:**
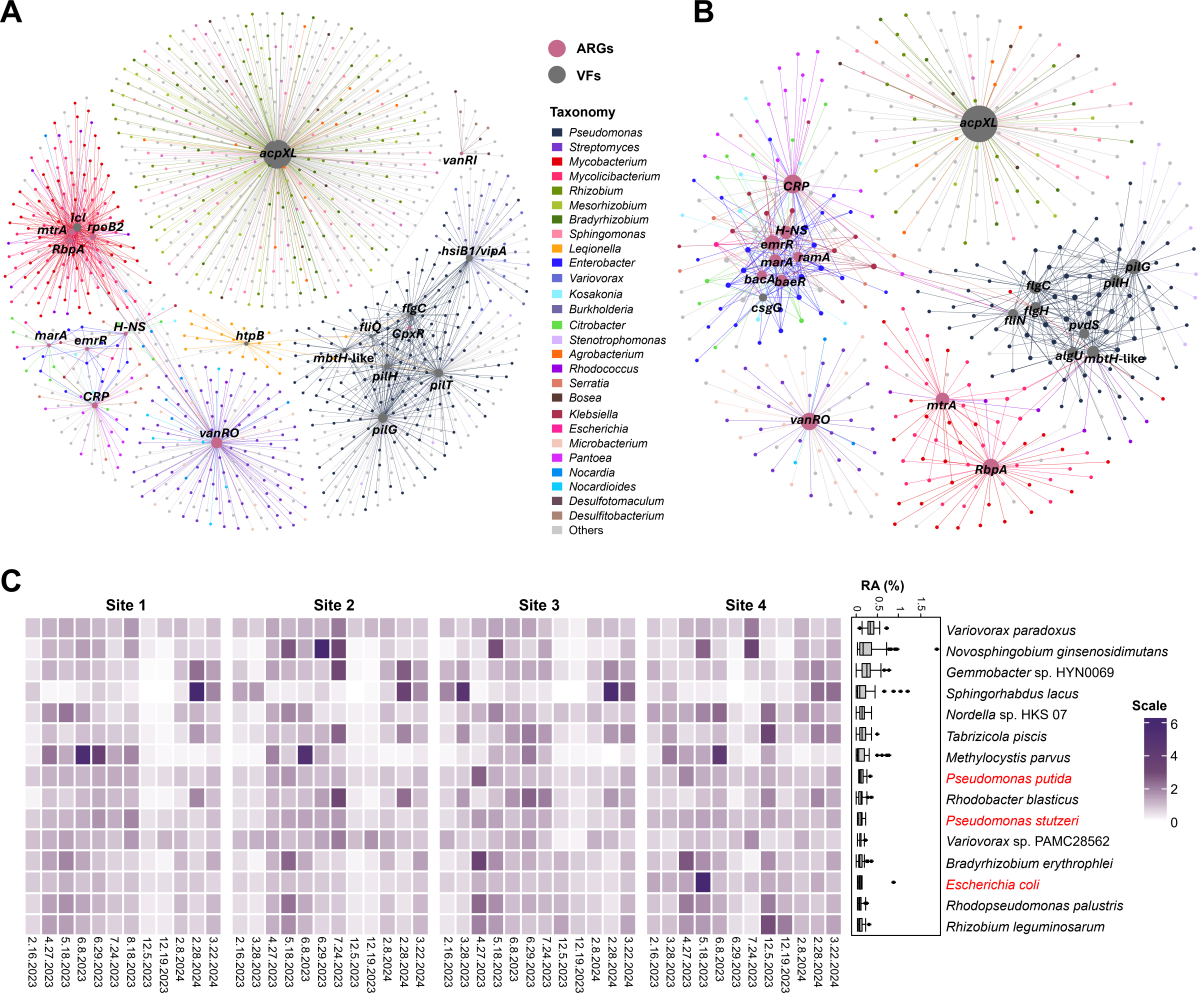
Network analysis illustrating the top 10 most abundant ARGs and VFs and their associated bacterial hosts, based on Nanopore long-read metagenomic sequencing data (**A**) and assembled Illumina contigs (**B**). Nodes represent ARGs, VFs, and microbial taxa, while edges indicate the relationships linking ARGs and VFs to the microbial taxa harboring them. The size of nodes represents their degree (i.e., number of connections). (**C**) Heatmap showing the normalized abundances of the top 15 most abundant bacterial species identified by Nanopore long-read metagenomic sequencing as carriers of the top 10 ARGs or VFs across the water samples. Normalization was performed by dividing the relative abundance by the average value across all 48 water samples. The accompanying box plot displays the average relative abundances for each species. Bacterial species labeled in red indicate opportunistic pathogenic strains included.

Network analysis showed that the 10 most abundant ARGs and VFs were distributed across a broad range of microbial taxa in the river water samples (Fig. 7A and B). A total of 1,175 species were identified as carrying at least one of the top 10 most abundant ARGs or VFs based on long-read metagenomics data (Fig. 7A), whereas 452 species were identified from the assembled Illumina contigs (Fig. 7B).

The networks revealed distinct distribution patterns of abundant ARGs and VFs across microbial taxa. Abundant ARGs were predominantly associated with genera such as *Streptomyces*, *Mycobacterium*, *Mycolicibacterium*, *Enterobacter*, *Klebsiella*, and *Microbacterium*, whereas abundant VFs were primarily linked to *Pseudomonas*, *Rhizobium*, *Mesorhizobium*, *Bradyrhizobium*, *Sphingomonas*, and *Legionella*. Some ARGs or VFs, such as *vanRO* and *acpXL*, were widely distributed across diverse bacterial taxa, whereas others, including *mtrA*, *RbpA*, *pilH*, *mbtH*, and *flgC*, were restricted to a narrow range of hosts (Fig. 7A and B). Additionally, several abundant ARGs, including *CRP*, *H-NS*, *marA*, and *emrR*, were detected across genera that encompass pathogenic species, such as *Enterobacter*, *Klebsiella*, *Escherichia*, and *Pantoea*. Furthermore, certain ARGs and VFs were found to co-associate with specific microbial taxa. For example, long-read metagenomics revealed that ARGs, such as *mtrA*, *rbpA*, and *rpoB2*, along with the VF *icl*, were frequently linked to *Mycobacterium* and *Mycolicibacterium* species. Likewise, seven of the ten most abundant VFs (*pilG*, *pilH*, *hsiB1/vipA*, *pilT*, *flgC*, *mbtH*, and *fliQ*), as well as the ARG *CpxR*, were primarily associated with *Pseudomonas* species (Fig. 7A). Consistent with this pattern, a contig from the assembled Illumina data, taxonomically classified as *Pseudomonas putida*, was detected by ABRicate to carry both the multidrug resistance-associated ARG *mexL* (an efflux pump regulator, multidrug resistance) and an *mbtH*-like VF (associated with siderophore biosynthesis). Notably, the network based on assembled Illumina contigs (Fig. 7B) displayed a highly similar pattern of host-ARG/VF associations to that observed in the ONT long-read-based network (Fig. 7A), suggesting the consistency of these associations across sequencing platforms.

Of the 1,175 species identified as carriers of top 10 abundant ARGs or VFs based on long-read metagenomics data, only 15 had a relative abundance exceeding 0.1% in the river water microbial communities (Fig. 7C). At Sites 1 to 3, these species generally exhibited higher relative abundances during spring and summer, but lower in winter, while Site 4 showed less seasonal variation (Fig. 7C). Among them, three species—*Pseudomonas putida*, *Pseudomonas stutzeri*, and *Escherichia coli*—include strains with known opportunistic pathogenic potential. Consistent with these findings, *P. putida* and *E. coli* were also among the top two species carrying the most diverse ARGs and/or VFs based on assembled Illumina data (Fig. S7).

## DISCUSSION

### Taxonomic resolution across sequencing methods

In our study, although amplicon, short-read, and long-read metagenomic sequencing of 48 river water samples revealed similar temporal trends in alpha diversity, metagenomic approaches provided higher species-level resolution. Short-read metagenomics identified over 2,000 species and long-read metagenomics over 3,000, compared to ~1,500 detected by amplicon sequencing. This is consistent with previous studies comparing amplicon and Illumina-based short-read metagenomics, which have shown that metagenomics is more effective at capturing low-abundance or underrepresented taxa (16, 40–42). The limited species-level resolution of amplicon sequencing is largely due to the high sequence similarity of variable regions within the 16S rRNA gene among closely related species and the biases introduced by PCR primers (43). Despite these limitations, amplicon sequencing performed well at higher taxonomic levels. In line with earlier findings (40, 41, 44), our results showed that amplicon data detected more phyla and genera than metagenomic methods.

All three sequencing methods detected Proteobacteria, Actinobacteria, Bacteroidetes, Cyanobacteria, and Firmicutes as the top five most abundant phyla in river water microbial communities. Interestingly, while a previous study reported comparable results between ONT long-read and Illumina short-read metagenomics at fine taxonomy levels (45), our results showed that long-read metagenomic data were more similar to amplicon data at the genus level, whereas short-read data diverged substantially. For example, abundant native freshwater planktonic genera widely reported in previous studies, such as *Polynucleobacter, Limnohabitans*, *Flavobacterium*, and *Rhodoluna* (46–49), were also among the most dominant taxa in both our amplicon and ONT long-read data sets. In contrast, Illumina short-read metagenomics detected these genera less frequently and instead emphasized genera containing potential pathogens, such as *Staphylococcus*, *Klebsiella*, *Acinetobacter*, and *Microbacterium*. However, the distinct taxonomic profiles identified by short-read metagenomics, as compared to amplicon data and long-read metagenomics, were unlikely due to contamination, as no taxa were detected in the negative control, and the taxa detected in the positive control matched its expected theoretical composition. Additionally, the same DNA extracts were used for both amplicon and Illumina short-read metagenomic sequencing, and the 16S rRNA gene data did not detect those divergent genera found in short-read metagenomics.

Notably, the amplicon and long-read metagenomic data sets, although based on DNA extracted by different laboratories using the same isolation kit, revealed highly similar bacterial community compositions. This aligns with previous studies showing that the use of standardized extraction kits can substantially reduce variability in microbial community profiles (50, 51). Furthermore, the consistency observed across platforms suggests that laboratory-specific DNA extraction bias was minimal in our study and reinforces the reliability of our conclusion that amplicon and long-read metagenomic approaches captured comparable microbial communities.

Additionally, although duplicate reads in Illumina shotgun metagenomics are often considered technical artifacts derived from library preparation and sequencing that may bias results (52, 53), removing them had minimal effect on microbial community structure and did not improve comparability with long-read metagenomics or amplicon results. Instead, the observed inconsistencies may stem from the limitations of Illumina’s short reads, since they are more likely to be misassigned to related taxa that are not actually present in the sample (54), particularly in k-mer-based classification tools like Kraken2. In contrast, ONT’s long reads can span repetitive or conserved regions that may lead to ambiguity in taxonomic assignments with shorter Illumina reads and provide more specific and accurate taxonomic assignments (45, 55, 56). Although Kraken2 was originally developed for short-read data, recent studies have demonstrated its effectiveness in classifying long-read sequences (57, 58). In our study, Kraken2 provided a consistent classification approach across sequencing platforms, and we applied abundance filtering to reduce the impact of low-confidence assignments.

### ARG and VF profiles across sequencing methods

Illumina short-read metagenomic sequencing detected greater diversity and normalized abundance of ARGs and VFs compared to ONT-based long-read metagenomics. Moreover, most ARGs and VFs identified by long-read metagenomics were also detected in short-read metagenomic data sets. Although no previous study has systematically compared the performance of ONT and Illumina platforms in profiling ARGs and VFs in aquatic ecosystems, one study reported comparable results between the two platforms in clinical blood samples (59). But in our study, long-read and short-read metagenomics showed distinct profiles of ARGs and VFs, with very limited overlap in the top 20 most abundant genes from each platform. The higher diversity and normalized abundance of ARGs and VFs detected in short-read metagenomics are likely driven not only by its higher sequencing depth and base accuracy, which increased the likelihood of capturing low-abundant or rare genes (60), but also by the nature of short-read sequencing. Specifically, the fragmentation of Illumina reads can inflate gene diversity and abundance estimates, as short reads may map to different regions of the same ARG or VF, and these partial hits may be counted as separate gene occurrences, leading to an overestimation of ARG or VF abundance. In contrast, ONT’s long reads—despite higher error rates—can span full gene sequences, reducing redundancy in detection and providing a potentially more accurate gene profile. Moreover, the observed differences between platforms may also be influenced by the alignment process. Illumina reads, with their high base accuracy, are more likely to pass the identity and coverage thresholds. Conversely, ONT’s long reads may fail to meet these thresholds due to sequencing errors or partial matches, leading to false negatives (61). It is worth noting that, although ARG and VF diversity largely decreased after assembly of Illumina reads, the profiles from assembled Illumina contigs aligned well with those from ONT long-read sequencing, especially for the genes of high abundance. This highlighted the trade-off between broader gene detection from unassembled Illumina reads and greater reliability from assembled contigs and also indicated the accuracy of long-read metagenomics in gene profiling (62).

Temporal dynamics of ARG and VF abundance in river water were effectively captured using long-read metagenomic sequencing, which showed elevated levels during winter and spring, with reductions in summer. These seasonal trends may be linked to precipitation-driven runoff, which introduces pollutants into aquatic ecosystems, including antibiotic-resistant bacteria and pathogens (63, 64). In contrast, spatial variation was more apparent in Illumina short-read data, particularly with higher levels of ARG and VF detected at Sites 3 and 4. These locations are more heavily influenced by anthropogenic activities, such as wastewater discharge, agriculture, and recreational use, which likely contribute to localized accumulation of antibiotic-resistant bacteria and pathogens (65, 66).

### Microbial host-ARG/VF associations

Overall, the microbial host-ARG/VF associations observed in ONT’s long reads and assembled Illumina contigs exhibited highly similar patterns. Among all detected ARGs, *vanRO* was the most abundant in both ONT long-read data sets and assembled Illumina contigs. It confers resistance to glycopeptides, such as vancomycin, and has been frequently detected in environmental samples, including soils and eutrophic water influenced by anthropogenic activities (67–69). In our study, *vanRO* was primarily associated with genera in the phylum Actinobacteria, especially *Streptomyces*, followed by *Microbacterium, Nocardia*, and *Nocardioides*, which is consistent with previous studies (69). While the *Streptomyces* species identified in our data sets are not known to be pathogenic, we also detected the occurrence of *Nocardia terpenica* and *Nocardia brasiliensis*, which have been reported as opportunistic pathogens (70, 71). However, their low abundance in the river water samples suggests a limited public health risk.

The most prevalent VF identified in both ONT long-read and assembled Illumina metagenomic data sets was *acpXL*, which was found in multiple genera, including *Rhizobium*, *Bradyrhizobium*, *Mesorhizobium*, *Brucella*, and *Brevundimonas*, consistent with previous studies (72, 73). *acpXL* facilitates biosynthesis of very long-chain fatty acids (VLCFAs) that modify lipid A, a key component of the bacterial outer membrane (74). This modification aids immune evasion of certain pathogens, such as some species of *Brevundimonas* and *Brucella* (75). However, in our results, *acpXL* was predominantly linked to rhizobia, which may be explained by that VLCFA-modified lipid A helping avoid recognition by plant immune systems, such as during root nodule formation, suggesting its non-pathogenic role in root colonization rather than a human health threat.

Notably, we observed co-occurrence of ARGs and VFs within individual microbial genomes. For example, several *Mycobacterium* species detected in the long-read metagenomic data carried both *rpoB2*, an ARG conferring rifampicin resistance, and *icl*, a VF encoding isocitrate lyase, which is essential for pathogen survival during infection via the glyoxylate cycle. While most of these species are not pathogenic to humans or animals, we also identified a strain closely related to *Mycobacterium canettii*, a member of the *Mycobacterium tuberculosis* complex (76), carrying both *rpoB2* and *icl*. Similarly, *Pseudomonas* species were prominent hosts of both ARGs and VFs, carrying multidrug resistance regulators, such as *cpxR* and VFs involved in adhesion, motility, and biofilm formation (*hsiB1*/*vipA*, *pilG*, *pilH*, and *pilT*). Although species, such as *Pseudomonas aeruginosa*, *Pseudomonas fluorescens*, *P. putida*, and *P. stutzeri,* are recognized as opportunistic pathogens (77–80), they were not dominant taxa in our samples.

ONT long-read metagenomic data showed that the opportunistic pathogens *P. putida* and *E. coli* carried several of the top 10 most abundant ARGs or VFs and had relative abundances exceeding 0.1%. Consistently, assembled Illumina data also identified them as the top species harboring the most diverse ARGs and/or VFs. Their higher prevalence at sites near urban areas highlights the influence of anthropogenic activity on the spread of potential antibiotic-resistant pathogens in freshwater ecosystems.

In conclusion, the advantages and limitations of ONT long-read and Illumina short-read metagenomic sequencing highlighted the value of hybrid approaches for balancing sensitivity, accuracy, and resolution in microbiome studies (81, 82). Illumina provides high sensitivity through deep coverage and base-level accuracy, whereas ONT’s long reads improve specificity and allow direct gene-host linkage. However, the cost of combining both platforms limits their feasibility for large-scale environmental applications. Our results showed that long-read metagenomics revealed genus-level microbial community composition, which closely matched amplicon data, more accurately captured dominant freshwater taxa than Illumina metagenomics, and generated comparable ARG and VF profiles, as well as microbial host associations to those from assembled Illumina data. Although assembled Illumina reads provided similar gene profiles, their low assembly rate in complex environmental metagenomes suggested a considerable loss of genetic information. Taken together, ONT-based long-read metagenomics represents an informative and reliable approach for high-resolution taxonomic and functional analysis of environmental microbiomes without the need for assembly. Additionally, our findings highlight the potential of metagenomic sequencing to enhance water quality monitoring by revealing antimicrobial resistance and pathogenic traits of microbial pollution, providing a more comprehensive assessment of microbial risks than traditional approaches based on fecal indicators and source tracking biomarkers.

## Data Availability

The sequencing data sets generated in this study have been deposited in the NCBI Sequence Read Archive (SRA). The 16S rRNA gene amplicon sequencing data are available under BioProject ID PRJNA1125451. The Illumina short-read metagenomic sequencing data are available under BioProject ID PRJNA1230144. The ONT long-read metagenomic sequencing data are available under BioProject ID PRJNA1126728.

## SUPPLEMENTAL MATERIAL

Supplemental material aem.01723-25-s0001.docx Tables S1 to S4; Fig. S1 to S7.

ASM does not own the copyrights to Supplemental Material that may be linked to, or accessed through, an article. The authors have granted ASM a non-exclusive, world-wide license to publish the Supplemental Material files. Please contact the corresponding author directly for reuse.
