# Supplementary material for: How sequencing technology shapes our understanding of river water microbiomes and resistomes: a comparative study
Source: Appl Environ Microbiol. 2025 Sep 19;91(10):e01723-25. doi: 10.1128/aem.01723-25 (PMC12542686; doi:10.1128/aem.01723-25)
Supplement: Supplemental material — Tables S1 to S4; Fig. S1 to S7. [file aem.01723-25-s0001.docx]

**Table S1**. Sampling information for Lavaca River water samples.

| Sampling Date | Site 1 | Site 2 | Site 3 | Site 4 |
| --- | --- | --- | --- | --- |
| 2023-2-16 | T1 | T1 | T1 | T1 |
| 2023-3-28 | - | T2 | T2 | T2 |
| 2023-4-27 | T2 | T3 | T3 | T3 |
| 2023-5-18 | T3 | T4 | T4 | T4 |
| 2023-6-8 | T4 | T5 | T5 | T5 |
| 2023-6-29 | T5 | T6 | T6 | T6 |
| 2023-7-24 | T6 | T7 | T7 | T7 |
| 2023-8-18 | T7 | - | - | - |
| 2023-12-5 | T8 | T8 | T8 | T8 |
| 2023-12-19 | T9 | T9 | T9 | T9 |
| 2024-2-8 | T10 | T10 | T10 | T10 |
| 2024-2-28 | T11 | T11 | T11 | T11 |
| 2024-3-22 | T12 | T12 | T12 | T12 |

**Table S2.** Summary of raw and clean reads and base pairs from 48 water samples (4 sampling sites × 12 sampling times) based on Nanopore long-read metagenomic sequencing and Illumina short-read metagenomic sequencing platforms.

| Sample  ID | Nanopore long-read metagenomics | | | Illumina short-read metagenomics | | |
| --- | --- | --- | --- | --- | --- | --- |
|  | # of raw reads | # of clean reads | # of bp in billion (clean reads) | # of raw reads | # of clean reads | # of bp in billion (clean reads) |
| S1-T1 | 9,332,109 | 9,281,766 | 31.02922 | 184,477,684 | 174,453,957 | 52.6851 |
| S1-T2 | 10,915,648 | 10,859,971 | 33.01831 | 154,443,908 | 143,205,331 | 43.24801 |
| S1-T3 | 10,429,363 | 10,419,726 | 32.23717 | 157,827,707 | 145,880,989 | 44.05606 |
| S1-T4 | 7,950,747 | 7,902,309 | 23.4569 | 160,003,345 | 146,487,678 | 44.23928 |
| S1-T5 | 6,949,963 | 6,906,939 | 20.54894 | 179,783,142 | 162,048,739 | 48.93872 |
| S1-T6 | 7,177,590 | 7,124,413 | 21.70815 | 275,016,935 | 255,594,640 | 77.18958 |
| S1-T7 | 7,208,536 | 7,171,294 | 23.04544 | 171,036,370 | 157,722,512 | 47.6322 |
| S1-T8 | 7,044,385 | 6,996,049 | 26.3945 | 181,583,480 | 167,260,648 | 50.51272 |
| S1-T9 | 8,612,582 | 8,544,602 | 29.77737 | 246,075,066 | 232,473,070 | 70.20687 |
| S1-T10 | 13,223,276 | 13,128,911 | 39.19425 | 145,407,230 | 134,361,826 | 40.57727 |
| S1-T11 | 9,527,118 | 9,453,776 | 28.18862 | 143,790,018 | 133,139,063 | 40.208 |
| S1-T12 | 12,092,783 | 12,000,357 | 33.75591 | 161,699,917 | 148,254,421 | 44.77284 |
| S2-T1 | 10,238,135 | 10,178,984 | 33.01235 | 189,119,261 | 174,084,556 | 52.57354 |
| S2-T2 | 11,076,016 | 11,022,045 | 38.24894 | 154,343,827 | 144,154,244 | 43.53458 |
| S2-T3 | 10,626,811 | 10,577,961 | 28.53669 | 179,351,794 | 166,596,593 | 50.31217 |
| S2-T4 | 15,753,582 | 15,676,314 | 47.1608 | 153,145,962 | 141,493,389 | 42.731 |
| S2-T5 | 6,370,748 | 6,335,371 | 20.52869 | 177,921,000 | 163,908,016 | 49.50022 |
| S2-T6 | 12,663,650 | 12,576,525 | 39.18885 | 133,970,651 | 122,074,329 | 36.86645 |
| S2-T7 | 8,470,281 | 8,400,344 | 25.94598 | 183,021,767 | 169,291,753 | 51.12611 |
| S2-T8 | 7,880,280 | 7,825,941 | 27.06283 | 158,183,583 | 147,742,584 | 44.61826 |
| S2-T9 | 13,357,781 | 13,288,916 | 45.04965 | 340,337,407 | 317,975,829 | 96.0287 |
| S2-T10 | 6,659,467 | 6,606,532 | 21.96507 | 119,082,198 | 111,415,030 | 33.64734 |
| S2-T11 | 10,028,844 | 9,952,719 | 28.7556 | 152,338,501 | 141,368,191 | 42.69319 |
| S2-T12 | 10,580,736 | 10,503,352 | 31.06078 | 169,989,171 | 156,659,187 | 47.31107 |
| S3-T1 | 10,340,078 | 10,283,428 | 31.76667 | 201,748,662 | 187,878,753 | 56.73938 |
| S3-T2 | 10,006,377 | 9,958,387 | 31.97289 | 192,268,747 | 177,586,000 | 53.63097 |
| S3-T3 | 11,126,534 | 11,052,665 | 28.55238 | 153,177,124 | 140,771,031 | 42.51285 |
| S3-T4 | 8,161,394 | 8,102,116 | 22.99813 | 156,886,115 | 146,161,605 | 44.1408 |
| S3-T5 | 8,274,775 | 8,220,731 | 27.14537 | 142,081,094 | 130,062,567 | 39.2789 |
| S3-T6 | 7,888,644 | 7,843,569 | 23.78196 | 220,002,271 | 202,664,777 | 61.20476 |
| S3-T7 | 10,455,024 | 10,384,050 | 31.67821 | 151,568,816 | 140,793,971 | 42.51978 |
| S3-T8 | 7,909,819 | 7,850,339 | 25.99922 | 130,548,897 | 121,381,057 | 36.65708 |
| S3-T9 | 7,309,769 | 7,250,279 | 27.84935 | 214,350,277 | 200,938,516 | 60.68343 |
| S3-T10 | 9,214,149 | 9,150,257 | 28.39949 | 211,800,247 | 196,014,373 | 59.19634 |
| S3-T11 | 11,231,273 | 11,143,128 | 30.9243 | 160,457,193 | 149,869,457 | 45.26058 |
| S3-T12 | 8,194,142 | 8,143,890 | 26.33863 | 127,693,928 | 117,438,434 | 35.46641 |
| S4-T1 | 8,093,721 | 8,031,998 | 27.93504 | 164,057,807 | 151,195,592 | 45.66107 |
| S4-T2 | 8,792,282 | 8,752,363 | 27.98034 | 127,243,194 | 118,698,485 | 35.84694 |
| S4-T3 | 9,913,967 | 9,829,622 | 26.84802 | 161,901,739 | 150,567,372 | 45.47135 |
| S4-T4 | 7,786,276 | 7,782,005 | 23.56076 | 155,160,509 | 143,144,942 | 43.22977 |
| S4-T5 | 6,659,133 | 6,617,423 | 21.21373 | 172,716,483 | 158,774,933 | 47.95003 |
| S4-T6 | 12,275,559 | 12,187,026 | 34.27465 | 212,472,696 | 195,792,200 | 59.12924 |
| S4-T7 | 12,814,599 | 12,732,771 | 39.33128 | 156,077,365 | 144,415,148 | 43.61337 |
| S4-T8 | 6,541,481 | 6,501,219 | 24.26753 | 187,972,160 | 173,673,074 | 52.44927 |
| S4-T9 | 7,223,538 | 7,171,057 | 25.24344 | 145,430,619 | 133,061,324 | 40.18452 |
| S4-T10 | 10,349,501 | 10,267,558 | 31.88312 | 149,193,577 | 137,353,774 | 41.48084 |
| S4-T11 | 8,523,381 | 8,459,658 | 24.10838 | 197,056,284 | 183,884,584 | 55.53314 |
| S4-T12 | 11,792,956 | 11,694,270 | 34.46962 | 162,626,065 | 150,224,610 | 45.36783 |

**Table S3.** Summary of assembly statistics of Illumina short-read metagenomic sequencing data.

| Sample ID | # of contigs | Average length (bp) | Max length (bp) | Total assembled bp | % assembled |
| --- | --- | --- | --- | --- | --- |
| S1-T1 | 1,032,984 | 966 | 115,865 | 998,167,272 | 1.89% |
| S1-T2 | 865,314 | 1,084 | 211,905 | 938,389,959 | 2.17% |
| S1-T3 | 957,275 | 854 | 60,633 | 818,346,608 | 1.86% |
| S1-T4 | 908,782 | 841 | 87,608 | 764,858,703 | 1.73% |
| S1-T5 | 1,000,281 | 915 | 119,254 | 916,164,292 | 1.87% |
| S1-T6 | 988,442 | 1,013 | 415,360 | 1,001,331,231 | 1.30% |
| S1-T7 | 1,282,093 | 1,110 | 229,947 | 1,423,451,568 | 2.99% |
| S1-T8 | 488,191 | 1,070 | 130,486 | 522,559,357 | 1.03% |
| S1-T9 | 706,785 | 1,091 | 403,806 | 771,475,333 | 1.10% |
| S1-T10 | 865,356 | 873 | 57,052 | 756,246,986 | 1.86% |
| S1-T11 | 976,472 | 962 | 87,875 | 939,859,113 | 2.34% |
| S1-T12 | 865,694 | 879 | 125,553 | 761,806,588 | 1.70% |
| S2-T1 | 1,099,140 | 931 | 126,500 | 1,023,646,983 | 1.95% |
| S2-T2 | 564,236 | 1,076 | 473,703 | 607,225,075 | 1.39% |
| S2-T3 | 1,165,099 | 888 | 145,243 | 1,035,508,567 | 2.06% |
| S2-T4 | 789,306 | 850 | 112,910 | 671,245,019 | 1.57% |
| S2-T5 | 957,294 | 953 | 1,111,326 | 912,620,853 | 1.84% |
| S2-T6 | 497,827 | 1,157 | 126,026 | 576,128,417 | 1.56% |
| S2-T7 | 738,685 | 1,103 | 238,698 | 815,498,900 | 1.60% |
| S2-T8 | 373,806 | 1,130 | 114,361 | 422,545,619 | 0.95% |
| S2-T9 | 1,133,879 | 1,006 | 349,685 | 1,141,804,101 | 1.19% |
| S2-T10 | 598,184 | 948 | 145,142 | 567,298,781 | 1.69% |
| S2-T11 | 627,926 | 911 | 106,435 | 572,055,218 | 1.34% |
| S2-T12 | 933,121 | 923 | 168,081 | 862,041,793 | 1.82% |
| S3-T1 | 1,095,983 | 1,012 | 146,124 | 1,109,546,643 | 1.96% |
| S3-T2 | 1,034,711 | 1,027 | 378,525 | 1,063,567,008 | 1.98% |
| S3-T3 | 653,781 | 779 | 49,882 | 509,418,858 | 1.20% |
| S3-T4 | 863,680 | 900 | 286,960 | 777,731,233 | 1.76% |
| S3-T5 | 799,725 | 924 | 96,537 | 738,986,859 | 1.88% |
| S3-T6 | 1,152,519 | 1,009 | 213,646 | 1,163,971,765 | 1.90% |
| S3-T7 | 643,895 | 1,034 | 240,696 | 666,309,416 | 1.57% |
| S3-T8 | 1,011,539 | 955 | 135,485 | 966,282,866 | 2.64% |
| S3-T9 | 939,436 | 1,062 | 312,857 | 998,206,150 | 1.64% |
| S3-T10 | 1,154,620 | 1,015 | 275,692 | 1,172,965,539 | 1.98% |
| S3-T11 | 800,887 | 1,073 | 173,778 | 859,931,696 | 1.90% |
| S3-T12 | 732,873 | 905 | 41,273 | 663,273,107 | 1.87% |
| S4-T1 | 1,016,611 | 950 | 122,774 | 966,498,333 | 2.12% |
| S4-T2 | 932,933 | 990 | 201,676 | 924,394,409 | 2.58% |
| S4-T3 | 859,168 | 857 | 54,805 | 737,092,245 | 1.62% |
| S4-T4 | 956,604 | 878 | 120,883 | 840,044,532 | 1.94% |
| S4-T5 | 992,019 | 968 | 126,001 | 961,227,116 | 2.00% |
| S4-T6 | 934,112 | 1,037 | 351,129 | 969,479,263 | 1.64% |
| S4-T7 | 593,684 | 1,075 | 526,616 | 638,373,301 | 1.46% |
| S4-T8 | 1,372,061 | 948 | 329,660 | 1,301,119,196 | 2.48% |
| S4-T9 | 563,756 | 1,031 | 300,335 | 581,752,645 | 1.45% |
| S4-T10 | 949,673 | 944 | 178,470 | 897,045,799 | 2.16% |
| S4-T11 | 1,164,628 | 988 | 321,400 | 1,150,837,153 | 2.07% |
| S4-T12 | 976,573 | 876 | 62,110 | 855,505,195 | 1.89% |

**Table S4.** *P*-values of Kruskal-Wallis test showing the effects of sampling site and sampling time on alpha diversity of river water samples based on different sequencing methods.

|  | 16S amplicon sequencing | | Nanopore long-read metagenomics | | Illumina short-read metagenomics | |
| --- | --- | --- | --- | --- | --- | --- |
|  | Site | Time | Site | Time | Site | Time |
| Observed species | 0.187 | 0.013 | 0.722 | 0.002 | 0.247 | 0.001 |
| Pielou’s evenness | 0.470 | 0.011 | 0.944 | < 0.001 | 0.037 | 0.010 |
| Shannon index | 0.375 | 0.012 | 0.885 | < 0.001 | 0.077 | 0.004 |


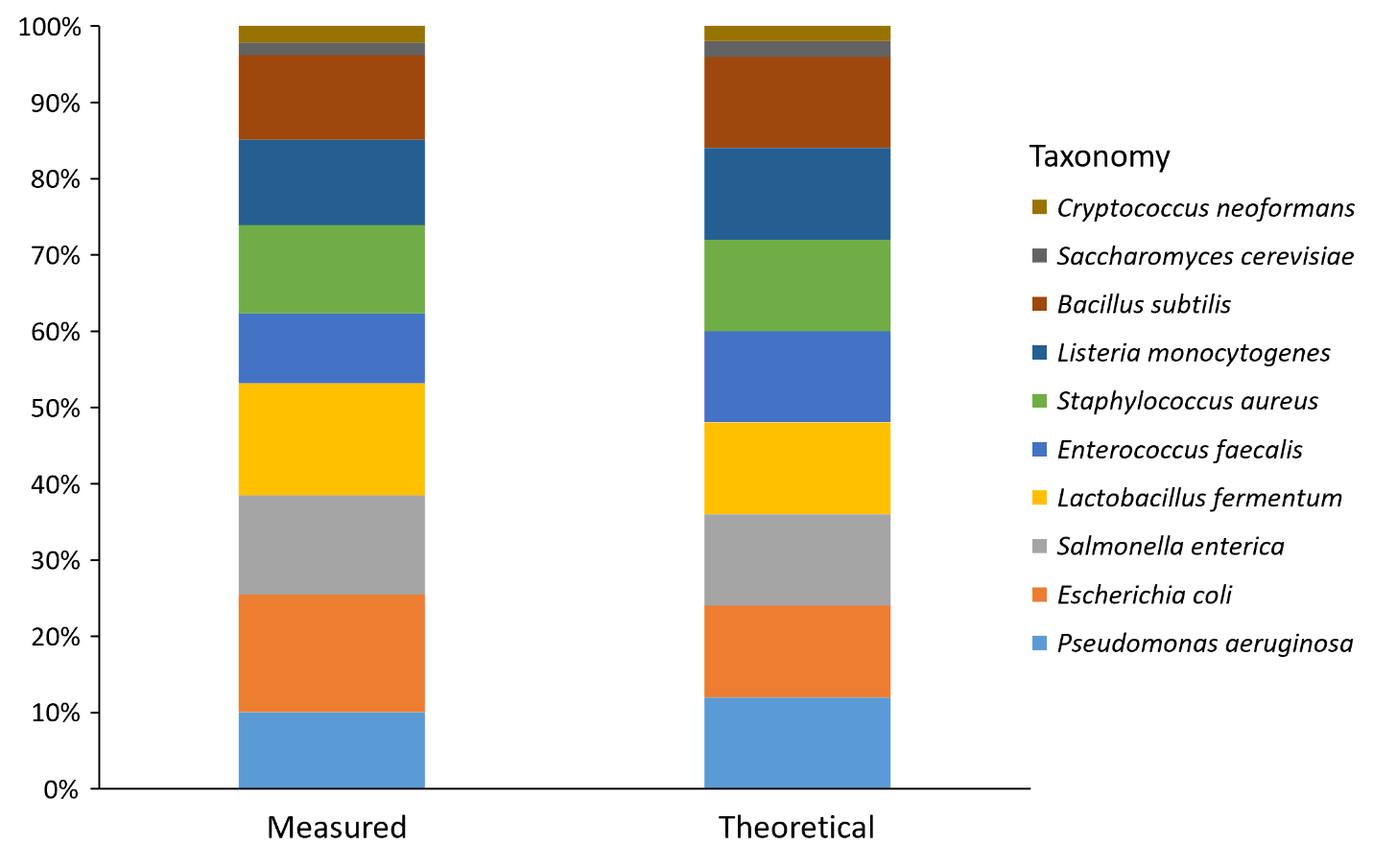


**Fig S1.** Comparison between the measured taxonomic profile and the theoretical composition of ZymoBIOMICS Microbial Community Standard used as a positive control.


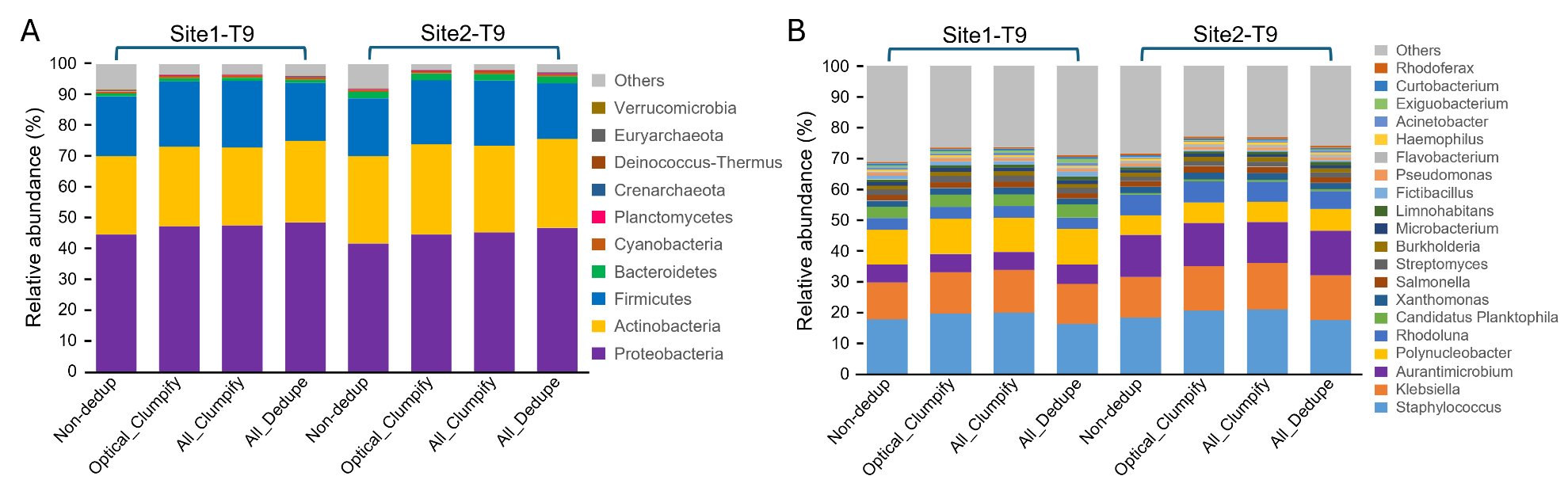


**Fig S2.** Taxonomic profiles of microbial communities in samples Site1-T9 and Site2-T9 before and after duplicate removal at phylum (**A**) and genus (**B**) levels. Non-dedup: no deduplication; Optical_Clumpify: optical duplicates removed by Clumpify; All_Clumpify: all duplicates removed by Clumpify; All_Dedupe: all duplicates removed by Dedupe.


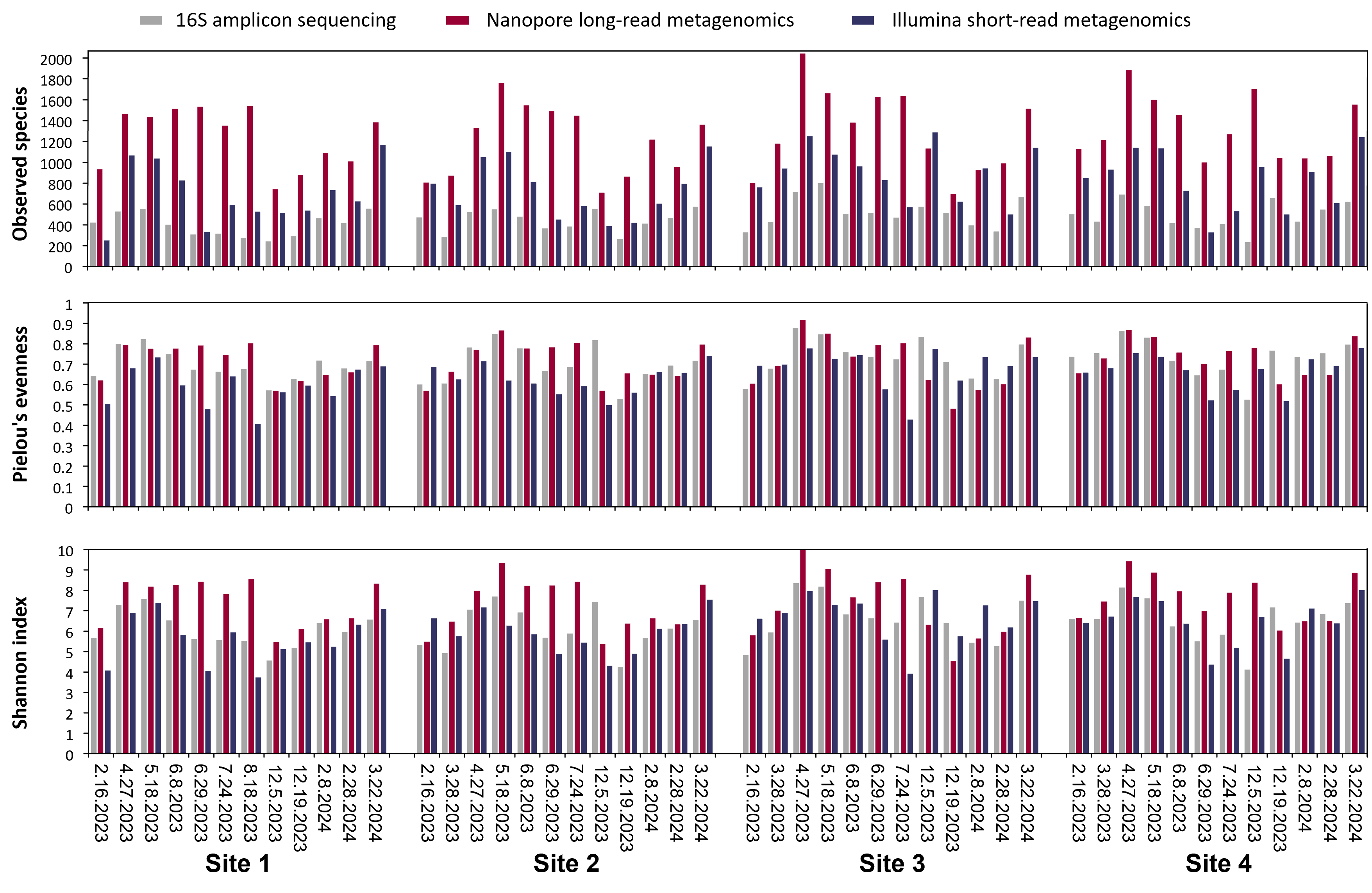


**Fig S3.** Alpha diversity of river water microbial communities based on 16S rRNA amplicon sequencing, Oxford Nanopore long-read metagenomic sequencing, and Illumina short-read metagenomic sequencing.


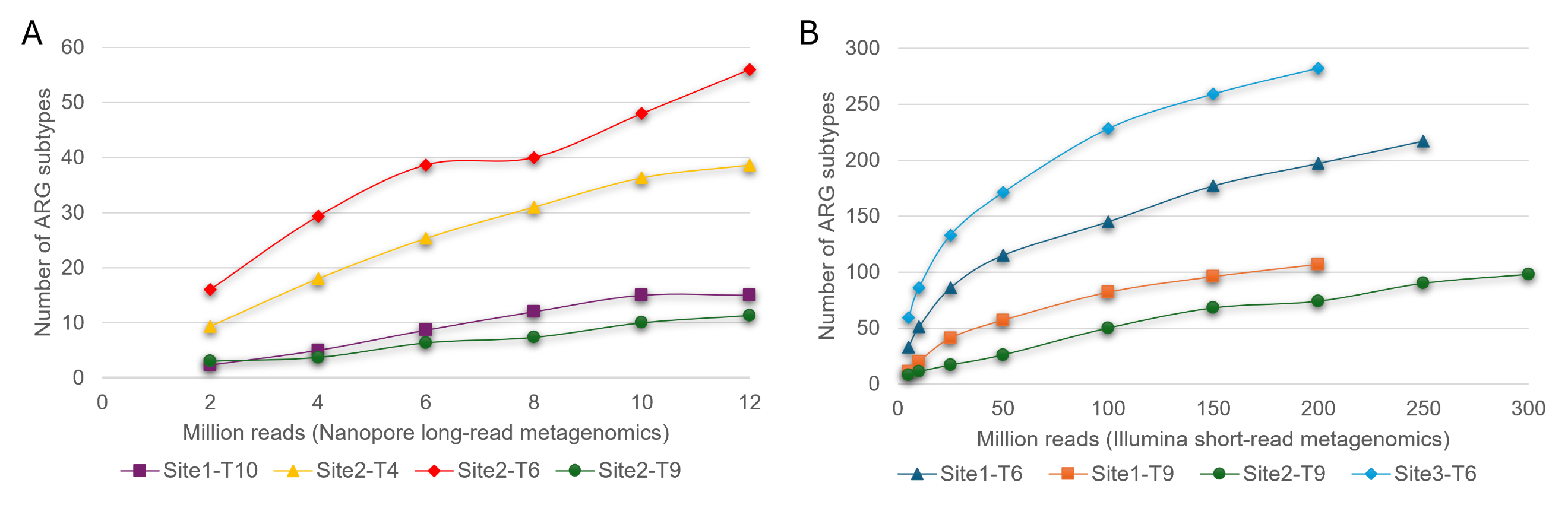


**Fig S4.** ARG-based rarefaction curves for the top 4 samples with the highest numbers of clean reads in Nanopore long-read (A) and Illumina short-read (B) metagenomic datasets.


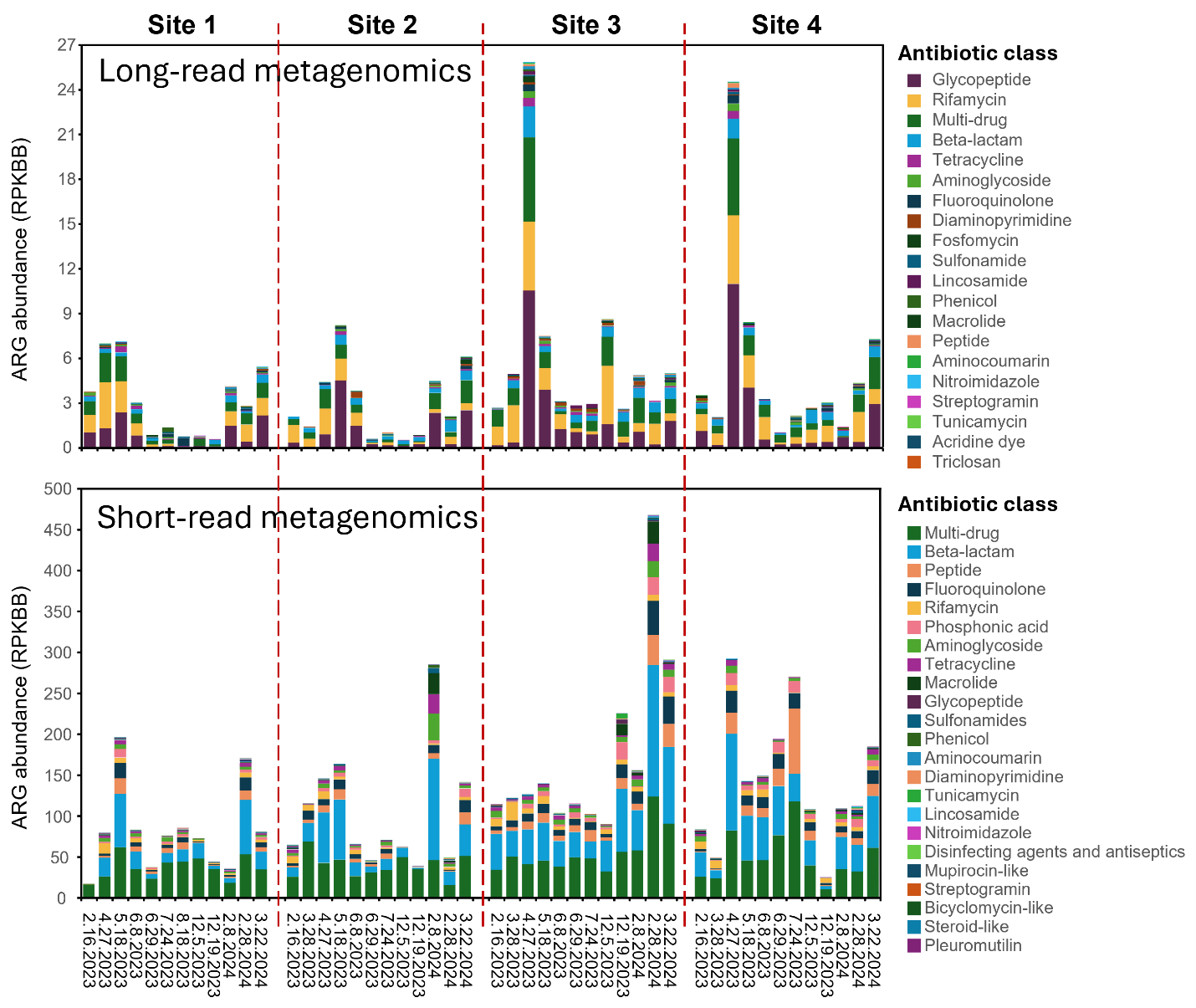


**Fig S5.** Normalized abundance of antibiotic resistance gene (ARG) types in river water samples detected by Nanopore long-read and Illumina short-read metagenomic sequencing.


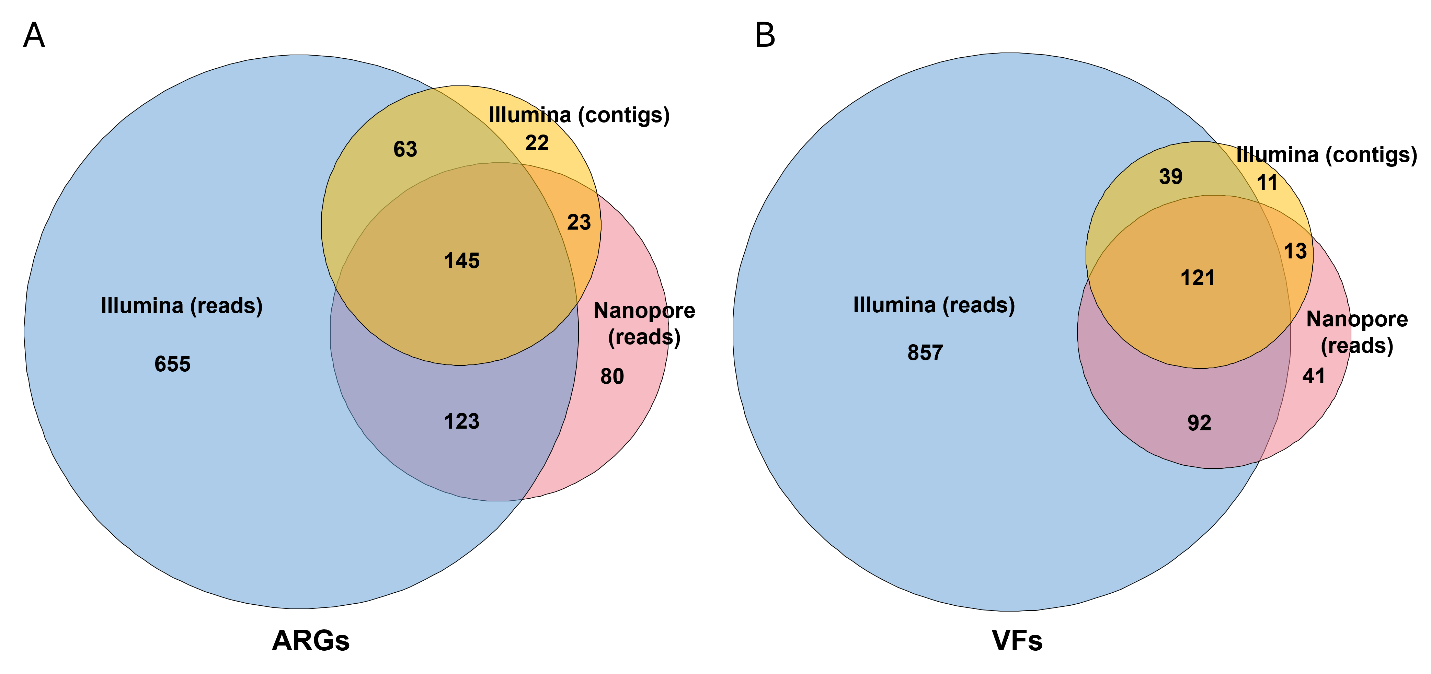


**Fig S6.** Venn diagrams illustrating the overlap of (**A**) antibiotic resistance genes (ARGs) and (**B**) virulence factors (VFs) detected in unassembled Illumina short reads, Nanopore long reads, and assembled Illumina contigs.


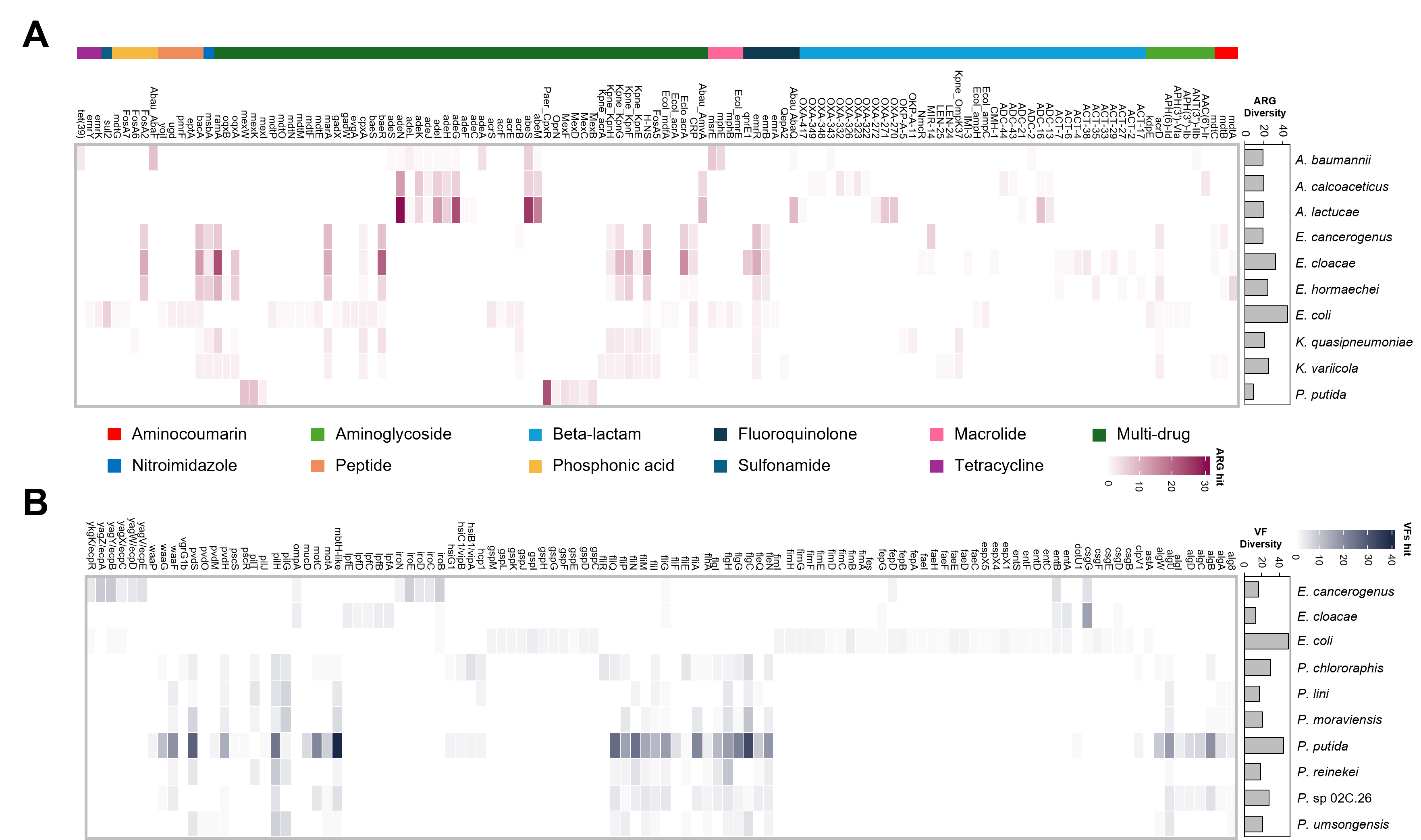


**Fig S7.** Heatmaps showing the distribution of ARG and VF gene counts across the top 10 bacterial species carrying the most diverse ARGs (**A**) and VFs (**B**). Each cell represents the number of distinct ARG or VF detected in a given species. The box plots show the number of unique ARGs or VFs detected per species, representing the diversity of gene types harbored by each taxon.
